# Multi-pathogen serological survey of migratory caribou herds: A snapshot in time

**DOI:** 10.1371/journal.pone.0219838

**Published:** 2019-07-31

**Authors:** A. M. Carlsson, P. Curry, B. Elkin, D. Russell, A. Veitch, M. Branigan, M. Campbell, B. Croft, C. Cuyler, S. D. Côté, L-M Leclerc, M. Tryland, I. H. Nymo, S. J. Kutz

**Affiliations:** 1 Faculty of Veterinary Medicine, University of Calgary, Calgary, Alberta, Canada; 2 Environment and Natural Resources, Government of the Northwest Territories, Yellowknife, Alberta, Canada; 3 CircumArctic Rangifer Monitoring and Assessment Network, Whitehorse, Yukon, Canada; 4 Department of Environment, Government of Nunavut, Iqaluit, Nunavut, Canada; 5 Greenland Institute of Natural Resources, Nuuk, Greenland; 6 Caribou Ungava, Département de Biologie and Centre d’études nordiques, Université Laval, Québec, Québec, Canada; 7 Department of Arctic and Marine Biology, UiT - The Arctic University of Norway, Tromso, Norway; 8 Canadian Wildlife Health Cooperative, Calgary, Alberta, Canada; University of Liverpool, UNITED KINGDOM

## Abstract

Pathogens can impact host survival, fecundity, and population dynamics even when no obvious disease is observed. Few baseline data on pathogen prevalence and diversity of caribou are available, which hampers our ability to track changes over time and evaluate impacts on caribou health. Archived blood samples collected from ten migratory caribou herds in Canada and two in Greenland were used to test for exposure to pathogens that have the potential to effect population productivity, are zoonotic or are emerging. Relationships between seroprevalence and individual, population, and other health parameters were also examined. For adult caribou, the highest overall seroprevalence was for alphaherpesvirus (49%, n = 722), pestivirus (49%, n = 572) and *Neospora caninum* (27%, n = 452). Lower seroprevalence was found for parainfluenza virus type 3 (9%, n = 708), *Brucella suis* (2%, n = 758), and *Toxoplasma gondii* (2%, n = 706). No animal tested positive for antibodies against West Nile virus (n = 418) or bovine respiratory syncytial virus (n = 417). This extensive multi-pathogen survey of migratory caribou herds provides evidence that caribou are exposed to pathogens that may have impacts on herd health and revealed potential interactions between pathogens as well as geographical differences in pathogen exposure that could be linked to the bio-geographical history of caribou. Caribou are a keystone species and the socio-economic cornerstone of many indigenous cultures across the North. The results from this study highlight the urgent need for a better understanding of pathogen diversity and the impact of pathogens on caribou health.

## Introduction

The Arctic is currently experiencing unprecedented climate change and anthropogenic disturbance that can influence the occurrence and spread of pathogens [1, 2]. Climate change has been linked to the emergence of diseases, escalating parasitic infection pressure, altered geographic distribution of pathogens and parasite invasions [3–5], whilst anthropogenic landscape modifications directly and indirectly impact the distribution and movement of host and vector species [1, 6]. Changes in exposure risk, emergence and spread of pathogens and disease in Arctic wildlife have already been observed [7–10]. Ecological perturbations arising from climate change have been linked to recent parasite range expansion, in, for example, muskoxen (*Ovibos moschatus)* and caribou [7] and widespread mortality events, in muskoxen [9], Saiga antelope *(Saiga tatarica tatarica)* [11] and reindeer [5].

Parasites and other pathogens can play key roles in ungulate population dynamics through direct or indirect effects on reproduction and survival (for example; [12–16]). They may also increase risk of predation [17, 18]. Establishing baselines of pathogen diversity is imperative to be able to understand the role of pathogens in individual and population health, and guide wildlife management and conservation [19–21]. From a One Health perspective this is particularly important at northern latitudes, where most people are dependent on harvested country foods (such as fish, waterfowl, caribou, moose, muskoxen, seals), and unhealthy animals can threaten human health, food security, and cultural well-being [22, 23].

Caribou (*Rangifer tarandus*) are an iconic keystone species in the circumpolar Arctic. They are important for ecosystem functioning and are the socio-economic cornerstone of many Indigenous cultures [24, 25]. During the last two decades, *Rangifer* populations have undergone substantial declines across their range, with climate change and environmental disturbance identified as contributors [26–28]. In Canada, the Committee on the Status of Endangered Wildlife in Canada (COSEWIC) has recommended that barren-ground caribou be listed as threatened [29] and that the Dolphin and Union herd, as well as the eastern migratory caribou in Quebec, be listed as Endangered [30, 31]. Pathogens and disease were identified in these assessments as potential threats to caribou population viability [29–31].

Serological surveys and other pathogen studies involving *Rangifer* began to enter the literature around the 1970s and reported prevalences of various pathogens that could affect herd and human health (for example; [32–35]). However, many herds remain unsampled or undersampled and baselines are still incomplete. Establishing pathogen diversity and prevalence is a critical first step for understanding trends and impacts of infectious disease in *Rangifer* and humans.

Obtaining samples needed to monitor pathogen prevalence in wildlife is both logistically and ethically challenging and subject to biases, as it often requires non-random capture, culling of the animal or locating carcasses, particularly in the case of elusive free-ranging wildlife in remote regions, such as caribou. Here, we used a unique collection of new and archived samples obtained in collaboration with government, scientists, local communities and harvesters across the North with two objectives: i) to describe the exposure of 12 migratory caribou herds/populations to eight pathogens that have the potential to impact herd health and productivity, are zoonotic, and/or are emerging (pesti-, herpes- and paramyxoviruses, *Neospora caninum*, *Brucella suis*, *Toxoplasma gondii* and West Nile Virus, Table 1), and; (ii) to examine relationships between seroprevalence and individual (age, sex), population (herd) and health (body condition, co-exposure) parameters.

**Table 1:** Pathogen impacts. For the pathogens screened for in this survey, known effects in *Rangifer* are listed when available, if effects are unknown effects in domestic animals are listed. The serological assays used were designed for bovine viruses and are likely cross-reacting with their cervid counterparts.

Agent	Type	Effects in *Rangifer*	Effects in domestic animals	Zoonotic impact
**Pestivirus**	Virus	Poorly studied. Loose bloody stools, laminitis[51]	Immunosuppression, respiratory and gastrointestinal disease, abortions, neonatal morbidity/mortality[115]	None
**Alphaherpes-virus (CvHV2)**	Virus	Oral lesions, infectious keratoconjunctivitis, pneumonia, abortion[49, 50, 96, 116]		None
**Paramyxo-viruses (PI3 and BRSV)**	Virus	Unknown	Contributes to Bovine respiratory disease complex[117]	None
***Neospora caninum***	Protozoan	Unknown	Abortions, mummified foetuses, weak calves[118]	None
***Brucella suis* biovar 4**	Bacteria	Abortion, weak calves, joint disease, orchitis, abscesses[40, 41]		Multi-systemic chronic disease[40]
***Toxoplasma gondii***	Protozoan	Abortion, lethal enteritis[119, 120]		Abortion, birth defects[46]
**West Nile virus**	Virus	Neurological disease, death[53]		Neurological, death[121] ^14^

## Materials and methods

### Sample collection

**Fig 1:**
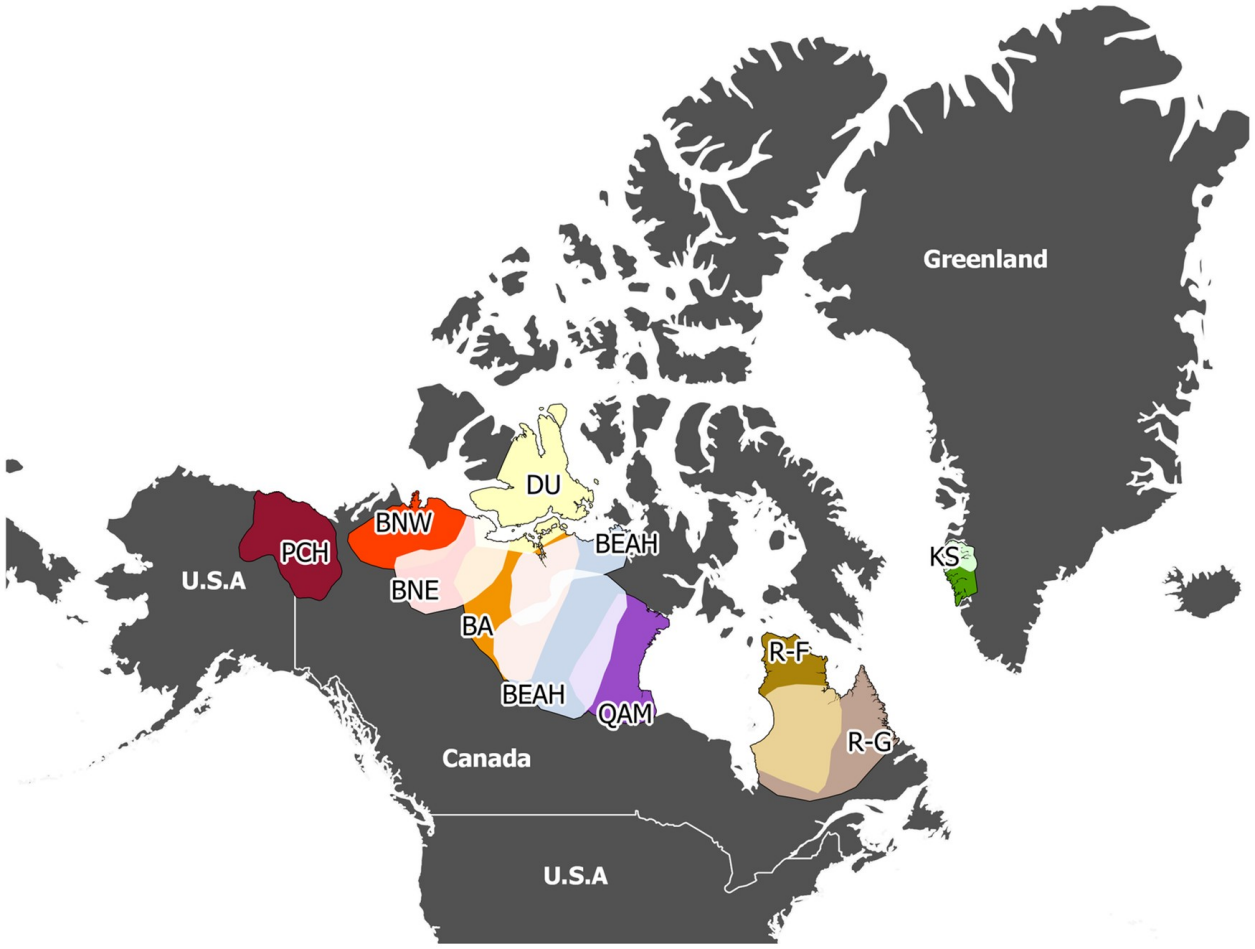
Ranges of the migratory caribou herds included in the serological survey. Porcupine (PCH), Bluenose-West (BNW), Bluenose-East (BNE), Dolphin and Union (DU), Bathurst (BA), Beverly and Ahiak (BEAH), Quaminuriaq (QAM), Rivière-aux-Feuilles (R-F), Rivière-George (R-G), Akia-Maniisoq (AK) and Kangerlussuaq-Sismiut (KA). Basemap sourced from Natural Earth (www.naturalearthdata.com) and caribou herd ranges from CARMA.

Samples were collected from the following migratory caribou herds and subspecies: Porcupine (PCH) (*R*. *t*. *granti*), Bluenose-West (BNW), Bluenose-East (BNE), Dolphin and Union (DU), Bathurst (BA), Beverly and Ahiak (BEAH), Quaminuriaq (QAM) (all *R*. *t*. *groenlandicus*), Rivière-aux-Feuilles (R-F), Rivière-George (R-G) (both *R*. *t*. *caribou*) in Canada, and the Akia-Maniitsoq (AK) and Kangerlussuaq-Sisimiut (KA) (*R*. *t*. *groenlandicus*) herds in Greenland (Fig 1 and S1 Table). Note that Beverly and Ahiak are recognised as two separate herds, but they could not be distinguished during the sampling and are, therefore, grouped together. During the International Polar Year (IPY), 2007–2009, the CircumArctic Rangifer Monitoring and Assessment network (CARMA), an international consortium of biologists, ecologists, aboriginal leaders, resource managers, veterinarians, and social scientists [36], coordinated an unprecedented collection of blood samples and health data from caribou herds across Canada and Greenland [37]. The majority of samples used in this study were from these collections. Additional samples were obtained in collaboration with local government agencies and subsistence hunters during community hunts, collaring events, community-based monitoring programs [23] and licensed guided hunts, and span a broader period (2000–2016). Sampling of herds was non-random and either directed by specific agency/research purposes, community interests or the needs of subsistence hunters. In general, community and subsistence hunts targeted presumably healthy animals, while the research collections were opportunistic and generally focused on adult females (and their calves for the R-F and R-G herds). Caribou of all ages and both sexes were sampled, but were not equally sampled among the herds or seasons (S1 Table). Samples came from a mixture of hunted and live-sampled animals that were captured during collaring projects.

Whole blood and/or filter-paper (FP) blood samples (Nobuto filter strips: Toyo Roshi Kaisha, Ltd., Tokyo, Japan) were obtained from each animal. Whole blood and FP samples were collected, stored and FP eluted as described in Curry, Elkin [38]. The exceptions were for R-F, R-G and DU herds, where blood was obtained during capture and collaring activities. For R-F and R-G samples, the blood was allowed to clot and separate, and the serum was drawn off and maintained in cool conditions until it could be transferred to a -20°C freezer. For the DU samples, freezing conditions in the field limited sample manipulation and blood was, in some cases, collected directly into a syringe and kept frozen at -20°C until it was transported to a laboratory where it was thawed, centrifuged and the serum drawn off.

Body condition data were collected according to methods outlined in CARMA level 1 and level 2 Monitoring protocols [37, 39]. Age classes (Calf (CA) < 1 year old, Yearling (YE) = 1 years old, Adult (AD) ≥ 2 years old) were assigned according to tooth cementum age analysis, (Matsons, Manhattan, MT, USA), where available, or based on classification determined in the field if incisors were not available.

### Pathogen selection rationale

Pathogens were selected based on previous knowledge of caribou exposure, relevance for herd and human health, and the availability of suitable serological assays (Table 1). In brief, *Brucella suis* biovar 4 is zoonotic and has been reported from all major barren-ground caribou herds from Alaska to Baffin Island but recent information on its prevalence and distribution is missing [40, 41]. *Neospora caninum* and *Toxoplasma gondii*, has previously been reported in *Rangifer* [42–45]. They can have reproductive impacts, which could lead to significant reductions in productivity and may additively influence population declines [16, 42]. *Toxoplasma gondii* can also cause disease in humans and seroprevalence in populations in Nunavik are up to three times higher (50–65%) than the North American average [46]. Exposure to herpes-, pesti- and paramyxo-viruses (detected through serological assays for Bovine herpes virus type 1 (BHV1, herpes virus), parainfluenza virus type 3 (PI3, paramyxovirus) and bovine viral diarrhoea virus (BVDV, pestivirus) has been recorded in *Rangifer*, whilst exposure to bovine respiratory syncytial virus (BRSV), or similar, has been recorded in other cervids [45, 47, 48]. In caribou, assays for these viruses are likely cross-reacting with cervid specific viruses. Cervid Herpes Virus 2 (CvHV2) was identified as the primary agent in an outbreak of keratoconjunctivitis in Norwegian reindeer [49] and experimental infections in reindeer have been linked to neonatal death and abortion [50]. The impacts of the other viruses (or similar cervid-specific viruses) are not well studied, although BVDV viremia in reindeer has been demonstrated by experimental infection [51]. In cattle, these viruses contribute to the bovine respiratory disease complex [52]. West Nile virus, a zoonotic pathogen that amplifies in avian hosts and is transmitted by mosquitoes, causes fatal disease in captive reindeer [53]. Range shifts of vectors in response to climate change could lead to the northward spread of this pathogen [54]

### Pathogen screening/testing

Samples were screened for antibodies to eight different pathogens or pathogen groups; alphaherpesvirus (Herpes), pestivirus (Pesti), parainfluenza virus type 3 (PI3), *Neospora caninum* (Neo), *Brucella suis* (Bru), *Toxoplasma gondii* (Toxo), West Nile virus (WNV) and bovine respiratory syncytial virus (BRSV). The specific tests used are summarized in Table 2.

**Table 2:** Tests and laboratories used for pathogen screening. Published validation and use in Rangifer or other cervids is indicated by references.

Pathogen^a^	Lab^b^	Test^c^	Kit	Cut-off^d^
Pestivirus	AUN	bELISA	SERELISA BVD p80 Ab Mono Blocking, Synbiotics Corp., France	P: >40%; D: 20–40%[55]
	CWHC	bELISA	IDEXX BVDV p80 Ab Test, IDEXX Laboratories Inc, Main, United States	P:≤40%; D:40 <S/N <50%
Alphaherpes virus	PDS	iELISA	In-house test using protein G-enzyme conjugate[122]	P: ≥14 EU
	AUN	bELISA	LSIVetT Bovine IBR gB Serum ELISA (based on BoHV-1 gB antigen)[91]	P: ≥35%
	AUN	bELISA	SERELISA IBR/IPV gB Ab Mono Blocking, Synbiotics, Europe SAS, France[91]	P: S/N <0.5 D: 0.55>S/N>0.5
PI3	PDS	iELISA	In-house test, adapted for cervids, protein G-enzyme conjugate[122], [56]	Serum: P: ≥14 EU FP: P: ≥10 EU[56]
*Neospora caninum*	AHC	cELISA	*Neospora caninum* Antibody Test Kit, cELISA; VMDR Inc., Pullman, WA, USA[56]	P: ≥30%
	PDS	cELISA	*Neospora caninum* Antibody Test Kit, cELISA; VMDR Inc., Pullman, WA, USA[56]	P: ≥30%
*Brucella*	BCE	cELISA	In-house testing based on antigen of *Brucella abortus*[38, 123],	P: ≥30%[69]
	AUN	iELISA	In-house testing based on a protein A/G iELISA[124]	P: ≥1.13%
*Toxoplasma gondii*	USDA	MAT[55, 125]		P: MAT titre ≥1:25
	CWHC	iELISA	IDSCREENToxoplasmosis indirect multispecies; IDvet., Grables, France	P: S/P% ≥50%; D: 40%< S/P%<50%
West Nile virus	PHA	cELISA	In-house testing using two monoclonal antibodies (mAb1, mAb2) [56, 126]	P: ≥30%
BRSV	PDS	iELISA	In-house test, adapted for cervids, protein G-enzyme conjugate[56, 122]	P: ≥14 EU

^a^ PI3 = Parainfluenza virus type 3; BRSV = bovine respiratory syncytial virus

^b^AUN = Research group of Arctic infection biology, Dept. of Arctic and Marine Biology, University of Tromso, the Arctic University of Norway; CWHC = Canadian Wildlife Health Cooperative, Alberta Node, Canada; PDS = Prairie Diagnostic Services, Saskatoon, SK, Canada; AHC = Animal Health Centre, Abbotsford, BC, Canada; BCE = Brucellosis Centre of Expertise, Ottawa, ON, Canada; USDA = United States Department of Agriculture, Parasite Biology and Epidemiology Laboratory, Beltsville, MD, USA; PHA = Zoonotic Diseases and Special Pathogens section, Public Health Agency of Canada, Winnipeg, MB, Canada

^c^ bELISA = Blocking Enzyme-Linked immunosorbent assay (ELISA); iELISA = Indirect ELISA; cELISA = Competitive ELISA; MAT = Modified Agglutination Test

^d^ P = Positive; D = Doubtful (suspect); % = % inhibition, calculated from OD (optical density) values, see Curry et al (2014); EU (ELISA Units) calculated from OD values, see Curry et al. (2014); S/N = OD sample/OD negative; %P (percent positivity) = ([OD sample/OD positive control]*100, see dasNeves (2009; S/P% = (OD sample-OD negative control/OD positive control-negative control)*100

Pathogen testing was performed in two rounds. First, samples collected during IPY from herds PCH, BA, R-F, R-G, AK and KA, and a subset of the BNW samples were tested between 2010 and 2011 [55]. Second, testing of additional samples obtained from archives or new collections from BNW, BNE, DU, BEAH, and QAM occured from 2014 and 2016. Due to limited sample volume, in some instances not all samples could be screened for all pathogens. In the first round, pathogen tests were prioritized as follows: Bru, Neo, WNV, Toxo, Herpes, BRSV, PI3, Pesti. Based on the results from the initial screening and the needs of collaborative projects, in the second round pathogen screening tests were not run for WNV or BRSV, and tests were prioritized as follows: Bru, Toxo, Herpes, Pesti, Neo and PI3.

FP eluates are estimated to be a 1:10 dilution of serum (Nobuto specifications: Toyo Roshi Kaisha, Ltd., Tokyo, Japan., Curry et al 2011). Thus, protocol steps were adjusted as needed to ensure that serum and FP results were comparable. The use of FP eluates in the place of serum was previously validated for the assays used in this study [38, 56]. As such, results from FP and serum samples were combined. The exception was for the *N*. *caninum* test (Table 2) where the kit uses undiluted serum. As such, no adjustments could be made to make the FP eluates, which are 1:10 serum dilution, comparable to the undiluted serum. Therefore, only results from *N*. *caninum* testing using serum samples are reported. Antibody tests were done at veterinary diagnostic laboratories in Canada, the United States and Norway. To the extent it was possible, we used assays that had been validated or tested in *Rangifer* (Table 2). Samples that fell within the range of suspect/doubtful values were re-run as per the manufacturers’ specifications (Table 2).

### Statistical analysis

For all analysis, samples that remained in the doubtful range after re-running (Table 3) were excluded since they could not be classified as seropositive or seronegative according to the threshold criteria. For *N*. *caninum* all analysis are based on results from serum samples, due to difficulties with the screening methodology for *N*. *caninum* from filterpaper samples.

**Table 3:** Adult seroprevalence. Observed sample seroprevalence of screened pathogens in adult caribou presented for female (F) and male (M) caribou, and overall (O) by herd. Pathogen abbreviations: Alphaherpesvirus (Herp), Pestivirus (Pesti), Parainfluenzavirus type 3 (PI3), *Neospora caninum* (Neo), *Brucella suis* biovar 4 (Bru), *Toxoplama gondii* (Toxo), West Nile Virus (WNV), Bovine respiratory syntical virus (BRSV). Caribou herd abbreviations: Porcupine (PCH), Bluenose West (BNW), Bluenose East (BNE), Dolphin and Union (DU), Bathurst (BA), Beverly and Ahiak (BEAH), Quaminuriaq (QAM), Rivière-aux-Feuilles (R-F), Rivière-George (R-G), Akia-Maniitsoq (AK) and Kangerlussuaq-Sisimiut (KA). Herds are listed west to east geographically left to right. Sample seroprevalence (%), number of positive samples (p), sample size (n), 95% Clopper-Pearson Exact confidence intervals (CI) and number of doubtful samples (D) are presented.

		PCH			BNW			BNE			DU	BA			BEAH	QAM			R-F	R-G	AK	KA	ALL HERDS		
		F	M	O	F	M	O	F	M	O	F	F	M	O	F	F	M	O	F	F	F	F	F	M	O
**Herp**	%	0	48	47	67	63	63	56	46	52	85	38	50	42	87	58	86	69	22	28	5	0	47	58	49
	CI	0–98	30–67	29–65	35–90	48–76	50–75	40–71	26–67	40–65	71–94	28–50	34–66	33–52	79–92	39–75	65–97	55–81	13–33	18–40	1–17	0–10	43–51	50–65	46–53
	p	0	15	15	8	32	40	24	11	35	35	30	20	50	105	19	19	38	16	21	2	0	260	97	357
	n	1	31	32	12	51	63	43	24	67	41	78	40	118	121	33	22	55	73	75	41	36	554	168	722
	D	0	0	0	0	0	0	0	0	0	1	0	0	0	0	0	0	0	0	0	0	0	1	0	1
**Pesti**	%	100	54	56	50	55	54	35	33	34	15	78	78	78	60	45	48	46	53	73	0	0	48	57	49
	CI	3–100	33–73	35–75	12–88	36–74	37–71	17–56	4–78	19–53	5–32	66–88	56–93	68–87	51–69	27–64	26–70	32–61	40–67	60–83	0–9	0–10	43–52	47–67	45–54
	p	1	14	15	3	16	19	9	2	11	5	47	18	65	68	14	10	24	31	45	0	0	223	60	283
	n	1	26	27	6	29	35	26	6	32	33	60	23	83	113	31	21	52	58	62	41	36	467	105	572
	D	0	0	0	6	10	16	17	18	35	2	19	17	36	8	2	1	3	15	13	0	0	82	46	128
**PI3**	%	100	45	47	0	12	10	31	13	25	0	5	0	3	5	10	0	6	12	5	0	0	8	14	9
	CI	3–100	27–64	29–65	0–26	4–24	4–20	18–47	3–32	15–36	0–9	1–13	0–9	1–8	2–11	2–26	0–15	1–16	6–22	1–13	0–9	0–10	6–10	9–20	7–11
	p	1	14	15	0	6	6	14	3	17	0	4	0	4	6	3	0	3	9	4	0	0	41	23	64
	n	1	31	32	12	51	63	45	24	69	37	78	40	118	118	31	22	53	73	75	41	36	547	168	715
**Neo**	%	0	0	0	0	0	0	-	-	-	22	1	3	2	68	81	81	81	0	0	0	0	29	19	27
	CI	0–98	0–15	0–15	0–98	0–22	0–21	-	-	-	10–38	0–7	0–15	0–6	59–76	63–93	58–95	67–90	0–12	0–13	0–9	0–10	24–33	12–29	23–31
	p	0	0	0	0	0	0	-	-	-	8	1	1	2	80	25	17	42	0	0	0	0	113	18	131
	n	1	22	23	1	15	16	-	-	-	37	75	36	111	118	31	21	52	28	28	41	36	396	94	490
**Bru**	%	0	0	0	0	4	3	2	0	1	15	5	5	5	0	0	0	0	0	0	0	0	2	2	2
	CI	0–98	0–11	0–11	0–25	1–10	1–9	0–12	0–14	0–8	6–29	1–12	1–17	2–11	0–3	0–11	0–15	0–6	0–5	0–5	0–9	0–10	1–4	1–6	1–3
	p	0	0	0	0	3	3	1	0	1	6	4	2	6	0	0	0	0	0	0	0	0	11	5	16
	n	1	31	32	13	84	97	43	24	67	41	80	40	120	121	33	22	55	73	75	41	36	557	201	758
**Toxo**	%	0	0	0	0	0	0	0	14	6	5	5	3	4	0	6	0	4	1	0	0	0	1	3	2
	CI	0–98	0–11	0–100	0–71	0–5	0–4	0–8	5–30	2–14	0–26	1–13	0–13	1–10	0–3	0–29	0–31	0–19	0–7	0–5	0–9	0–10	1–3	1–7	1–3
	p	0	0	0	0	0	0	0	5	5	1	4	1	5	0	1	0	1	1	0	0	0	7	6	13
	n	1	31	32	3	80	83	46	35	81	19	79	40	119	121	17	10	27	73	74	41	36	510	196	706
	D	0	0	0	0	0	0	1	2	3	1	0	0	0	0	0	0	0	0	0	0	0	2	2	4
**WNV**	%	0	0	0	0	0	0	-	-	-	-	0	0	0	-	-	-	-	0	0	0	0	0	0	0
	CI	0–98	0–11	0–11	0–98	0–9	0–8	-	-	-	-	0–5	0–9	0–3	-	-	-	-	0–5	0–5	0–9	0–10	0–1	0–3	0–1
	p	0	0	0	0	0	0	-	-	-	-	0	0	0	-	-	-	-	0	0	0	0	0	0	0
	n	1	31	32	1	41	42	-	-	-	-	79	40	119	-	-	-	-	73	76	41	36	306	112	418
**BRSV**	%	0	0	0	0	0	0	-	-	-	-	0	0	0	-	-	-	-	0	0	0	0	0	0	0
	CI	0–98	0–11	0–11	0–98	0–9	0–8	-	-	-	-	0–5	0–9	0–3	-	-	-	-	0–5	0–5	0–9	0–10	0–1	0–3	0–1
	p	0	0	0	0	0	0	-	-	-	-	0	0	0	-	-	-	-	0	0	0	0	0	0	0
	n	1	31	32	1	41	42	-	-	-	-	78	40	118	-	-	-	-	73	76	41	36	306	112	417

The observed sample seroprevalence of each pathogen was calculated for each herd for adults, yearlings and calves and for males and females (where possible) based on the pathogen screening results. 95% confidence-intervals were calculated using epitools epidemiological calculators [57] employing the Clopper-Pearson exact method, as it produces conservative intervals with reduced risk of over-estimating seroprevalence [58].

For Pesti, Herpes and Neo we examined factors influencing seropositivity and the relationship between seropositivity and body condition. This analysis was not possible to perform for other pathogens due to quasi complete separation of the data [59]. Data from the AK and KA caribou populations were excluded from further statistical analyses because, with only two exceptions (two samples from AK that tested positive for alphaherpesvirus) all samples were seronegative for all pathogens.

To maximise sample size and avoid data separation [59] we used two approaches for analysis, employing three subsets of data. First, we examined factors influencing seroprevalence of Pesti, Herpes and Neo using generalized linear models (GLMs) with a binomial (logit) link. Pesti and Herpes models were fitted to a subset of data containing results from samples that had been tested for exposure to both pathogens (Pesti and Herpes) (n = 569). Neo models were fitted using a subset of data containing results from serum samples that had been tested for exposure to Pesti, Herpes and Neo and was restricted to the three herds for which Neo seroprevalence was > 10% (DU, BA and BEAH). Explanatory variables of interest were: age class (as determined by field observations), Sex, Co-exposure (Herpes and/or, esPesti serostatus (positive, negative)), and Herd (PCH, BNW, BNE, DU, BA, BEAH, QAM, R-F, R-G). Separate models were run for each pathogen. Due to an unbalanced dataset and limited sample size of certain subsets, the only interaction included was between Sex and Co-exposure for Herpes and Pesti analysis. Models were fit using the *glm* function from the “stats” package in R [60].

Second, we tested whether seropositivity to Pesti and/or Herpes predicted caribou body condition using linear models. Two different indices of body condition were used as the response variable in separate models: Riney kidney fat index, calculated as the ratio of the weight of the kidney fat to the weight of the kidney * 100 (KFI), and direct measures of back fat in millimeters (mm)[39]. For KFI, we used a subset of samples comprising non-pregnant adult females from R-F and R-G herds collected in summer (June-July) and fall (October-November) for analysis (n = 119). For back fat, we used the same subset of data, but restricted it to animals sampled in fall (n = 58), since back fat of animals sampled in summer measured 0 mm. Explanatory variables included Tage (as determined by cementum age analysis), Tage^2, Year (2007, 2008, 2009), Pesti and Herp serostatus (positive, negative). No interactions were fitted due to the limited sample size. Herd (R-F, R-G) was included in all models to account for baseline differences in body condition between the herds. For models predicting KFI, Season (summer, fall) was also included in all models to account for seasonal variations in body condition. Models were fit using the *lm* function from the “stats” package in R [60].

Candidate models were created including different combinations of biologically plausible explanatory variables, and compared using Akaike’s Information Criterion with a second-order correction for small sample sizes (AIC_c_) [61]. Among the top models with a ΔAIC_c_ < 2, the simplest model was selected as the best fit if the other top model(s) only differed by one parameter from the model with the lowest AIC, and had a minimal reduction in log-likelihood (i.e., they did not improve explanatory power), indicating that those additional parameters were uninformative [62]. For the first approach examining seroprevalence, eighteen different models were tested for Pesti and Herpes and 31 models for Neo. For the second approach examining body condition 24 models were run for backfat and 24 for kidneyfat.

### Ethics statement

This study was carried out in strict accordance with the recommendations in the Guidelines of the Canadian Council on Animal Care and the relevant Federal and Provincial legislation in such a manner to minimise suffering. Protocols were approved by the University of Calgary Animal Welfare Committee (protocol numbers BI-2006-52 BI 2007–52, BI2008-45, BI08R-45, AC13-0121). Samples were received from animals killed by hunters for food, biologist/wildlife officers or researchers for other projects or from live captured animals. All sampling required adherence to standardized and approved protocols for sampling or killing wildlife species, aimed at reducing stress and suffering of the animals. All captured caribou were captured by professional capture crews under the standard operating procedures of partnering agencies.

## Results

### Seroprevalence

Results for observed sample seroprevalence are presented in Table 3. The number of samples tested varied by pathogen due to limited blood sample volume (see Methods). After re-testing, 128 samples screened for pestivirus were still classified as doubtful. Results discussed below are from adults. Seroprevalence results for yearlings and calves can be viewed in S2 and S3 Tables.

Overall seroprevalence for adults was, by far, the highest for alphaherpesvirus (49%, CI: 46–53, range: 0–87%, n = 722) and pestivirus (49%, CI: 45–54, range 0–78%, n = 572). All herds except for the two Greenland caribou herds (although see comment below and Discussion) were seropositive for these two pathogens. *Neospora caninum* had the third highest seroprevalence (based on serum samples only; 27%, CI:23–31%, range:0–81%, n = 490) followed by PI3 with a seropositivity of 9% (CI: 7–11, range: 0–47%, n = 715) (Table 3). Seropositivity for *B*. *suis* was 2% (CI: 1–3, range: 0–5%, n = 758), with individuals from BNW, BNE, DU and BA herds testing positive. Seropositivity for *T*. *gondii* was 2% (CI:1–3, range: 0–7%, n = 706), with individuals from BNE, DU, BA and QAM testing positive. All samples were negative for WNV (n = 418) and BRSV (n = 417). The two Greenland caribou herds (KA and AK) were seronegative for all pathogens, with the exception of two alphaherpesvirus-positive samples from the AK herd.

Evidence for exposure to more than one pathogen was assessed in a subset of data where each individual had been tested for exposure to all pathogens (excluding results from KS and AK and pathogen testing for WNV and BRSV) and classified as either seropositive or seronegative (n = 474, doubtfuls removed). Two hundred and forty (46%) individuals had been exposed to more than one of the tested pathogens, 84 (16%) individuals were seropositive for three different pathogens, and three individuals (0.6%) from BEAH and one individual from QAM (0.2%) were seropositive for four different pathogens.

### Factors associated with exposure to pestivirus

Two models predicting Pesti seropositivity were within a ΔAIC_c_<2, we based our inference on Model 1p (see Methods and Table 4).

**Table 4:** Models predicting seroprevalence. Summary of top 10 models models, based on ΔAICc, predicting alphaherpesvirus (Herp), pestivirus (Pesti) and Neospora caninum (Neo) seroprevalence. K is the number of parameters, wi is the model Akaike weight and LL is the log likelihood. The model used for inference is highlighted in grey.

Pathogen	Models	K	AIC_c_	ΔAIC_c_	w_i_	LL
Pesti	1p. Herd+Age+Herp	12	698.31	0.00	0.62	-336.88
	2p. Herd+Age+Sex+Herp	13	699.93	1.62	0.27	-336.64
	3p. Herd+Age+Sex+Herp+Sex:Herp	14	701.77	3.46	0.11	-336.51
	4p. Herd+Age	11	713.56	15.25	0	-345.54
	5p. Herd+Age+Sex	12	715.56	17.25	0	-345.50
	6p. Herd+Herp	10	719.59	21.28	0	-349.60
	7p. Herd+Sex+Herp	11	719.89	21.57	0	-348.71
	8p Herd+Sex+Herp+Sex:Herp	12	721.96	23.65	0	-348.70
	9p. Herd	9	747.48	49.17	0	-364.58
	10p. Herd+Sex	10	748.53	50.21	0	-364.07
Herp	1h. Herd+Age+Se+Pesti	13	590.15	0.00	0.49	-281.75
	2h. Herd+Age+Se+Pesti+Sex:Pesti	14	591.34	1.19	0.27	-281.29
	3h. Herd+Age+Pesti	12	591.53	1.37	0.24	-283.48
	4h. Herd+Age+Sex	12	605.29	15.14	0.00	-290.36
	5h. Herd+Age	11	606.47	16.32	0.00	-292.00
	6h. Herd+Pesti	10	618.32	28.17	0.00	-298.96
	7h. Herd+Sex+Pesti	11	618.57	28.42	0.00	-298.05
	8h. Herd+Sex+Pesti+Sex:Pesti	12	619.97	29.82	0.00	-297.70
	9h. Herd	9	646.21	56.06	0.00	-313.94
	10h. Herd+Sex	10	647.03	56.88	0.00	-313.32
Neo	1n. Herd+Herp+Pesti	5	230.53	0.00	0.31	-110.11
	2n. Herd+Pesti	4	231.19	0.66	0.23	-111.49
	3n. Herd+Sex+Herp+Pesti	6	232.46	1.94	0.12	-110.02
	4n. Herd+Sex+Pesti	5	233.23	2.70	0.08	-111.46
	5n. Herd+Age+Herpes+Pesti	7	233.37	2.85	0.08	-109.40
	6n. Herd+Age+Pesti	6	233.56	3.03	0.07	-110.56
	7n. Herd+Age+Sex+Herpes+Pesti	8	235.35	4.83	0.03	-109.31
	8n. Herd+Age+Sex+Pesti	7	235.67	5.14	0.02	-110.55
	9n. Herd	3	236.13	5.60	0.02	-115.00
	10n. Herd+Herpes	4	237.47	6.94	0.01	-114.63

**Fig 2:**
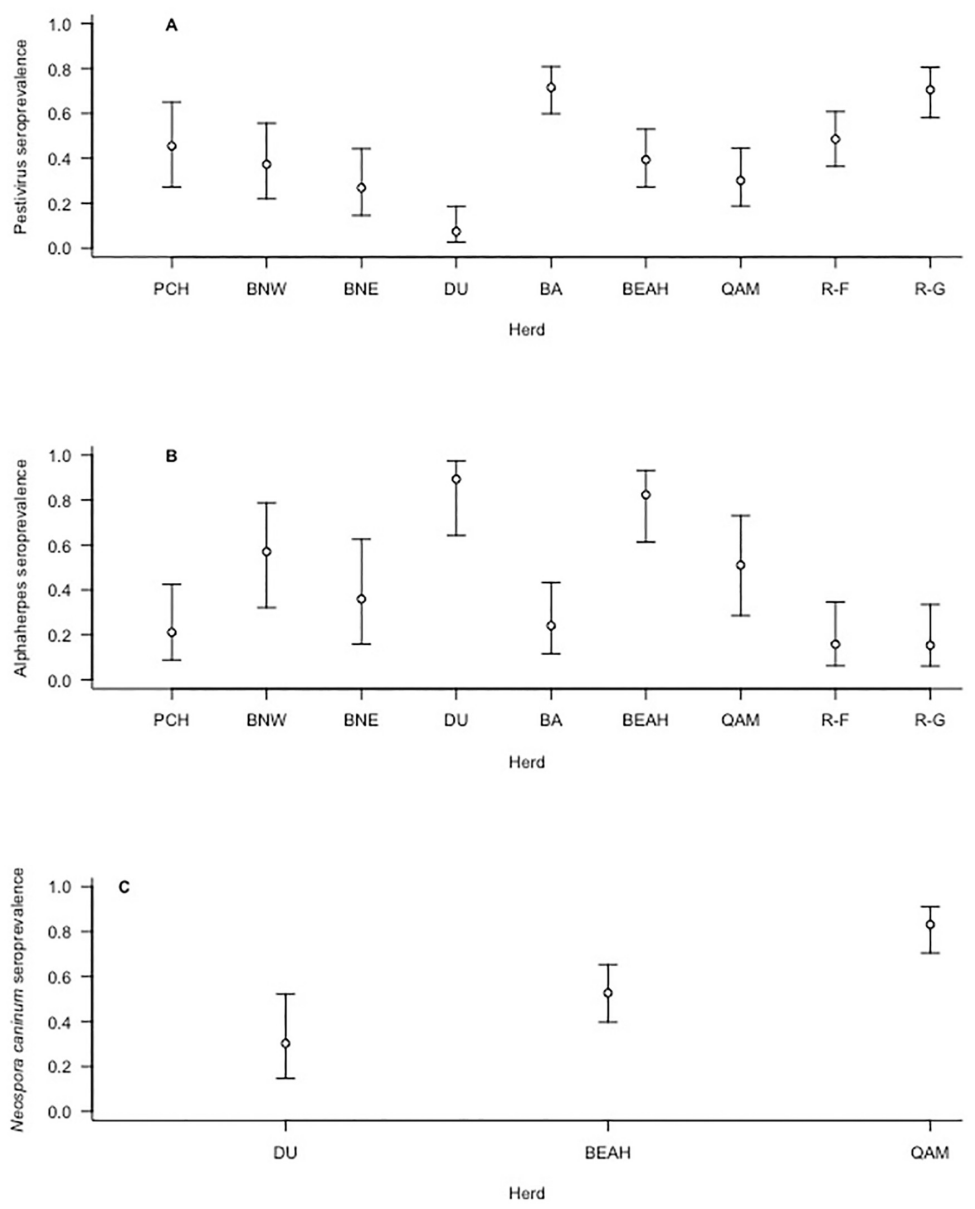
Herd differences in seroprevalence for pestivirus (A) herpesvirus (B) and *Neospora caninum* (C). Herds are listed from west to east; Porcupine (PCH), Bluenose-West (BNW), Bluenose-East (BNE), Dolphin and Union (DU), Bathurst (BA), Beverly and Ahiak (BEAH), Quaminuriaq (QAM), Rivière-aux-Feuilles (R-F), Rivière-George (R-G) Figure shows predictions for adult caribou, without co-infection, based on the selected model (Table 4).

There was variation in seroprevalence for Pesti among herds, with R-G and BA having the highest predicted seroprevalence and DU the lowest (Fig 2A). Adults were more likely to be seropositive for pestivirus relative to calves (Odds ratio (OR) = 2.4, CI = 1.2–4.6) and yearlings (OR = 10.2, 95% CI = 3.4–44.3). Calves were more likely to be seropositive relative to yearlings (OR = 4.3, 95% CI = 1.2–20.9). Animals that were seropositive for Herpes were more likely to be seropositive for Pesti than herpes negative animals (OR = 2.6, 95% CI = 1.6–4.1).

### Factors associated with exposure to alphaherpesvirus

There were three models within a ΔAIC_c_<2 predicting Herpes seropositivity, we based our inference on model 3h (see Methods and Table 4).

There was variation in seroprevalence for Herpes among herds, but this differed from the pattern for pestivirus. For alphaherpesvirus, DU and BEAH had the highest predicted seroprevalence and R-G and R-F had the lowest predicted seroprevalence, closely followed by PCH and BA (Fig 2B). Similar to Pesti, adults were more likely to be seropositive for alphaherpesvirus relative to calves (OR = 12.3, 95% CI = 3.6–77.8) and yearlings (OR = 4.5, 95% CI = 1.9–11.4). When estimating the risk of seropositivity for calves relative to yearlings (OR = 0.4, 95% CI = 0.05–1.7), the yearlings appeared at higher risk, but the 95% confidence intervals overlapped one suggesting the difference may be negligible.

Co-exposure predicted Herpes seropositivity. Compared to Pesti-negative animals, those that were seropositive for Pesti were more likely to also be seropositive for Herpes (OR = 2.6 95% CI = 1.6–4.1).

### Factors associated with exposure to *N*. *caninum*

There were three models within a ΔAIC_c_<2 predicting Neo seropositivity, we based our inference on model 2n (see Methods and Table 4).

There was variation in seroprevalence for Neo among the three herds tested, where QAM had the highest predicted seroprevalence followed by BEAH and DU (Fig 2C). In addition, Pesti seropositivity predicted Neo seropositivity. Compared to Pesti-negative animals, those that were seropositive for Pesti were more likely to also be seropositive for Neo (OR = 2.5, 95% CI = 1.3–5.0).

### Body condition

Within a ΔAIC_c_<2 there was support for three models predicting kidneyfat and two predicting backfat, we based our inference on Model 3k (explanatory variables: Herd, Tage and Season) and Model 1b (explanatory variable: Herd) (S4 Table). Neither model included Pesti or Herpes as predictors of bodycondition.

## Discussion

This study represents an unprecedented geographic scope of sampling and pathogen testing of northern caribou herds and improves our understanding of pathogen diversity and exposure in caribou. It demonstrates that caribou are exposed to pathogens that are important for herd health, some of which are zoonotic and can be detrimental to human health.

### Herd differences in exposure

There was a notable absence of seropositivity to the tested pathogen groups in the samples from the two Greenland herds (KA and AK) and, with the exception of Herpes and Pesti, seroprevalence was low overall in the Quebec and Labrador herds (R-F and R-G). Herd was an important covariate for predicting both alphaherpesvirus and pestivirus seroprevalence, although no distinct pattern could be discerned. Differences among herds and regions can have arisen as a result of ecological, demographic, behavioural and evolutionary factors [32, 63, 64] but sampling regimes may also have influenced results.

Historical biogeography could explain some differences in observed seroprevalence among herds. The sampled herds are descended from different lineages; the western Canadian barren-ground caribou originate from the Beringian-Eurasian lineage and the Quebec-Labrador herds from the North American Lineage [65]. Greenland was colonized by one or several populations of barren-ground caribou descending from the Beringian-Eurasian lineage [66, 67]. The loss of pathogens during these colonization events was hypothesized as the explanation for the unexpectedly low diversity of gastrointestinal parasites found in KA and AK herds [63]; this founder effect may also explain the similarly low pathogen biodiversity detected with serology in these herds. A serosurvey of alphaherpesvirus, pestivirus and PI3 in Svalbard reindeer, inhabitants of the similarly isolated archipelago Spitsbergen in Norway, were also all seronegative [68].

With respect to pathogen diversity in the Quebec and Labrador herds, differences in the parasitological fauna compared to barren-ground caribou have previously been described and likely link back to their historical biogeography [32, 64]. Similar differences in pathogens detected through serology are also expected. The serological assays used here are not specific to *Rangifer* viruses, rather to bovine viruses and to a broader taxonomic level of viruses. This means that while we observe seropositivity to, for example, alphaherpesvirus and pestivirus, it does not mean that it is the same virus circulating in both the Quebec and Labrador herds and the Beringian lineage herds. Rather, it is possible that distinct groups of pathogens have remained circulating within and among the populations that descended from these distinct lineages, and these pathogens may have divergent life-history and transmission dynamics that are reflected by differences in the seroprevalence. However, since serological assays work by detecting antibodies, that persist for variable lengths of time in the blood and provide evidence of past exposure to a pathogen, it is difficult to conclusively derive information on the timing, intensity and frequency of infection from serological data and link that information to environmental or demographic changes [69]. To fully understand and confirm herd differences in pathogen exposure, further studies within a shorter and more synchronised time-span with a well stratified sampling regime are needed.

### Reproductive limiting pathogens

The comparatively high seropositivity for three reproduction limiting pathogens, *Toxoplasma gondii* (5%), *Brucella suis* (15%) and *N*. *caninum* (22%), in the Dolphin and Union caribou herd is of particular note. The Dolphin and Union samples were collected in 2015 and 2016, coinciding with an ongoing population decline [31]), A local knowledge study reported fewer juveniles, more animals in poor body condition, and more frequent sightings of limping caribou with swollen joints during this period [70]. Subsequently the herd status was recommended to be changed from Special Concern to Endangered by COSEWIC [23].

Poor reproduction, bursitis and lameness are symptoms commonly associated with infection with *B*. *suis* biovar 4 in caribou [71] A previous study on caribou of Southampton Island demonstrated a substantial population decline associated with increasing seroprevalence of *B*. *suis* biovar 4 [72] Notably, with the exception of the high seroprevalence in the DU herd (15%), seroprevalence in the remaining herds (0–5%) was lower than reported in historical studies (ranging between 9–40%) [33, 73–75].

Caribou seropositive to *T*. *gondii* and *N*. *caninum* have been reported across the Arctic and Subarctic North America, with prevalence ranging from 0.7–62.5% and 1.4–15.7%, respectively [76]. These apicomplexan parasites are transmitted both through predator-prey linkages and vertically from mother to foetus [77]. Exposure risk is linked to the presence and density of definitive hosts (canids for *N*. *caninum* and felids for *T*. *gondii* [78]). There are no definitive hosts for these parasites on Greenland, thus, absence of seropositivity to both these parasites in AK and KA caribou herds is not surprising.

In comparison to previous surveys of *N*. *caninum*, there was an unexpectedly high seroprevalence for QAM (81%), BEAH (68%) and DU (22%) caribou, whilst few or no seropositive samples were detected in the other Canadian herds tested. The reason for these differences remain unclear but could be linked to geographical differences and the presence and density of definitive hosts [78]. Our analyses showed that animals seropositive for pestivirus were more than twice as likely to also be seropositive for *N*. *caninum*. Associations between seropositivity for *N*. *caninum* and bovine diarrhoea virus have been detected in cattle in some [79, 80] but not all studies [81]. It has been suggested that concurrent *N*. *caninum* and pestivirus infections aggravate disease and abortion risk in cattle, but studies have shown conflicting results [82, 83]. *Neospora caninum* was implicated as the cause of widespread fetal mummification and loss in at least one captive reindeer herd [42]), however, the impacts of single or co-infections in caribou populations is unknown. The high seroprevalence of this parasite in declining caribou herds highlights the urgent need to better understand the consequences of this parasite in caribou productivity, and the association with potentially shifting predator-prey interactions.

### Alphaherpesvirus and pestivirus

The high seroprevalence for alphaherpesvirus and pestivirus in all herds (with the exception of KA and AK) is in accord with most previous serological surveys for these viruses in *Rangifer* worldwide [49, 68, 75, 84–90]. Although the assays used in this study detected antibodies reacting against antigens of bovine viral diarrhea virus (BVDV) and bovine herpesvirus type 1 (BHV-1), they also cross-react with antibodies to cervid-specific viruses [91, 92]. The lack of direct contact between domestic ruminants and *Rangifer* further suggests an independent infection process with cervid-specific viruses [93]. An alphaherpesvirus, designated cervid herpesvirus 2 (CvHV2), has been isolated from reindeer on multiple occasions [49, 50, 94–96]. It is likely that it is this virus to which the caribou in our study are reacting. In contrast, pestivirus has not been isolated from free-ranging *Rangifer*, but a distinct pestivirus was isolated from a reindeer (*R*. *t*. *tarandus*) in a German Zoo [92].

We observed a strong effect of age on risk of exposure to both pestivirus and alphaherpesvirus, with adult caribou the most likely to be exposed in both cases. This is consistent with previous *Rangifer* studies [85, 87, 90, 97], and not unexpected since older animals have had a longer period of potential exposure to the virus. In addition, alphaherpesvirus can establish latency and may be re-activated under stressful conditions. Reactivation will boost production of antibodies and increase the likelihood of a positive serological result [47]. For pestivirus (but not alphaherpesvirus), calves were more likely to be exposed compared to yearlings, however, antibodies detected in calves may have been maternal antibodies. It is currently unknown how long pestivirus antibodies persist in *Rangifer*; in cattle, maternal antibodies can be detected in calves for up to 6 months, after which they are at risk of infection [98]. The majority (67%) of the caribou calves in our study were 4–5 months old. Cattle can experience transplacental transmission of BVDV, and calves infected during the first trimester of pregnancy develop immunotolerance and are born persistently infected (PI), yet seronegative. These PI animals serve as an ongoing source of infection for their herd mates and can result in a high seroprevalence and low herd productivity [99]. Whether this phenomonen of PI animals occurs in *Rangifer* is unknown and is a critical issue to be determined in order to understand the epidemiology and impacts of this pathogen across the herds. Although sex was not an informative parameter in predicting pestivirus or alphaherpesvirus seroprevalence, further studies are needed to conclusively determine whether there are sex differences in exposure risk. Previous *Rangifer* studies have reported conflicting results [85, 88, 97].

Caribou seropositive for alphaherpesvirus were more than twice as likely to also be seropositive for pestivirus, and vice versa. Such an interaction was observed in a serological survey of semi-domesticated reindeer in Sweden [87] and co-infections have been noted in reindeer in Norway [100], but our findings are the first evidence of this interaction in wild caribou. BVDV and BHV-1 infection in cattle are both associated with immunosuppression and may predispose infected individuals to secondary infections [101, 102]. Furthermore, infection with BVDV may lead to enhanced virulence and pathogenesis of secondary infections [103]. Experiments in bovine calves have shown that animals previously infected with BVDV are less effective at containing infection with BHV-1 and present with more severe clinical signs, and presence of BHV-1 favours BVDV persistence [104–106]. If similar processes occur in *Rangifer*, this could explain the observed pattern. Research in pathogen-host interactions has been dominated by the study of “one-host-one-pathogen systems”, especially in wildlife [107]. However, in nature, most animals are infected with multiple pathogens, and evidence from field studies and models show that interactions between pathogens can be critical for the dynamics and virulence of infection, as well as for pathogen management [108–110].

Whereas BVDV and BHV-1 infection has been associated with reduced weight gains in cattle [102, 111], we were unable to detect an association between exposure to alphaherpesvirus or pestivirus and caribou body condition. However, the samples used for analysis were not collected to explicitly test for associations between exposure and fitness; therefore, they had limited power to detect effects [112]. As such, our results should not be interpreted as evidence that there is no effect; rather, they highlight the need for studies aimed at determining the impact of these pathogens on *Rangifer* health.

### Other seroprevalence findings

Although surveys for PI3 exposure in caribou have been limited, similar to our study, exposure has been detected in some, but not all, *Rangifer* herds tested [33, 84, 89]. No samples tested positive for WNV or BRSV. Exposure to BRSV, or similar, has been recorded in other cervids [47, 48] but no cases are reported in the literature for *Rangifer* spp. This may reflect absence of the pathogen, or more likely, that the Bovine RSV test is highly specific to the bovine strain and doesn’t cross-react with a *Rangifer* specific RSV [113]. WNV has been reported to cause fatal disease in captive reindeer [53] but has not been detected in free-ranging *Rangifer* to date. Range shifts of vectors in response to climate change could lead to the northward spread of WNV [54]. The samples tested in our study provide a baseline of exposure, and future monitoring can help determine whether they will become an emerging threat.

Serological surveys are fraught with challenges yet can still yield important results to inform on pathogen diversity and disease ecology. We recognize several limitations of this study. Samples were not collected with the explicit purpose of disease surveillance and, in the case of subsistence hunter-harvested animals, the sampling was biased towards healthy individuals. Furthermore, the absence of species-specific assay validation and cross-reactivity of antibodies can have implications for sensitivity, specificity and cut-off values of assays [69] and these complicate data interpretation. To address these issues, where possible, we employed assays that had been validated for use in *Rangifer* or other cervid species. We also aimed for consistency in the laboratories and assays used for testing. The time period from which samples were collected is also relatively large. Due to the unknown, but potential differences in seroconversion over-time, a narrower window would be preferential to make more robust comparisons, detect temporal patterns and make inferences of exposure among herds. Recognizing these limitations, and the relatively small sample size per herd in relation to herd population size, our analysis still revealed important patterns of exposure worthy of further investigation. The sample and analysis limitations also mean that the detected seroprevalence levels may underestimate the true population-level exposures.

## Conclusions

*Rangifer* across their range have undergone severe and prolonged population declines, and although natural population cycles, environmental and anthropogenic disturbance and habitat alteration have been implicated as possible causes, the reasons for the declines remain enigmatic [26–28]. Several of the pathogens to which antibodies were detected may have significant impacts on reproduction and health. Unfortunately, our data did not allow us to test for associations between exposure, reproduction or recruitment, but our results clearly show that further investigation of the disease ecology of the pathogens surveyed here, and other infectious agents in caribou, is warranted. The vast geographic scope of this survey was only possible due to the large-scale collaborations and diligently archived samples facilitated by the CARMA network and by the historical and ongoing efforts of numerous Inuit and First Nations harvesters, government biologists, and academics. These types of collaboration, where samples and expertise are shared across disciplines, can provide invaluable knowledge and results for science and management in fields where resources are scarce.

Our study demonstrates that several pathogens of concern are circulating in migratory caribou populations. To better understand the role of these pathogens in caribou population dynamics, there is need to isolate and identify the viruses circulating in *Rangifer* and to implement longitudinal studies and experiments designed to evaluate and anticipate impacts of these pathogens on caribou population dynamics. Additionally and more generally this study highlights the need for species-specific standardized diagnostic tests for wildlife pathogens. Importantly, in this study we only tested for what we thought may exist–the unknown pathogens, of which we are certain there are many (e.g. Kutz et al 2015 [9]), were not investigated. The rapid advancement of new molecular methodologies and genomic approaches will hopefully make extensive surveys for the unknowns possible in the near future [114].

## Supporting information

S1 Table Sample collection. Summary of seasons and years sample collection occurred for each herd, including the type of collection. CARMA IPY refers to scientific collections conducted during the International Polar Years by the CircumArctic Rangifer Monitoring Assessment Network.
(PDF)

S2 Table Seroprevalence for yearlings. Observed sample seroprevalence of screened pathogens for yearling female (F) and male (M) caribou, and overall (O) by herd. Pathogen abbreviations: Alphaherpesvirus (Herp), Pestivirus (Pesti), Parainfluenzavirus type 3 (PI3), *Neospora caninum* (Neo), *Brucella suis* biovar 4 (Bru), *Toxoplama gondii* (Toxo), West Nile Virus (WNV), Bovine respiratory syntical virus (BRSV). Caribou herd abbreviations: Porcupine (PCH), Bluenose West (BNW), Bluenose East (BNE), Dolphin and Union (DU), Bathurst (BA), Beverly and Ahiak (BEAH), Quaminuriaq (QAM), Rivière-aux-Feuilles (R-F), Rivière-George (R-G), Akia-Maniitsoq (AK) and Kangerlussuaq-Sisimiut (KA). Herds are listed west to east geographically left to right. Sample seroprevalence (%), number of positive samples (p), sample size (n), 95% Clopper-Pearson Exact confidence intervals (CI) and number of doubtful samples (D) are presented.
(PDF)

S3 Table Seroprevalence for calves. Observed sample seroprevalence of screened pathogens for calf female (F) and male (M) caribou, and overall (O) by herd. Pathogen abbreviations: Alphaherpesvirus (Herp), Pestivirus (Pesti), Parainfluenzavirus type 3 (PI3), *Neospora caninum* (Neo), *Brucella suis* biovar 4 (Bru), *Toxoplama gondii* (Toxo), West Nile Virus (WNV), Bovine respiratory syntical virus (BRSV). Caribou herd abbreviations: Porcupine (PCH), Bluenose West (BNW), Bluenose East (BNE), Dolphin and Union (DU), Bathurst (BA), Beverly and Ahiak (BEAH), Quaminuriaq (QAM), Rivière-aux-Feuilles (R-F), Rivière-George (R-G), Akia-Maniitsoq (AK) and Kangerlussuaq-Sisimiut (KA). Herds are listed west to east geographically left to right. Sample seroprevalence (%), number of positive samples (p), sample size (n), 95% Clopper-Pearson Exact confidence intervals (CI) and number of doubtful samples (D) are presented.
(PDF)

S4 Table Top 10 models predicting kidney fat index and backfat in caribou. Summary of top 10 models, based on ΔAIC_c_, predicting kidney fat index and backfat in caribou, where K is the number of parameters, w_i_ is the model Akaike weight and LL is the log-likelihood. The model used for inference is highlighted in grey. Explanatory variables included were: Herd, Season (Summer, Fall), Tage (age as determined by cementum tooth age analysis), Sex, Co-exposure (alphaherpesvirus (Herp) and/or, esPestivirus serostatus) (positive, negative)) and sampling year.
(PDF)

S1 File Data file. Minimal dataset.
(XLSX)

## References

[1] Gottdenker NL, Streicker DG, Faust CL, Carroll C. Anthropogenic land use change and infectious diseases: A review of the evidence. EcoHealth. 2014;11(4):619–32. 10.1007/s10393-014-0941-z 24854248

[2] Dudley JP, Hoberg EP, Jenkins EJ, Parkinson AJ. Climate change in the North American Arctic: a one health perspective. EcoHealth. 2015;12(4):713–25. 10.1007/s10393-015-1036-1 26070525

[3] Kutz SJ, Jenkins EJ, Veitch AM, Ducrocq J, Polley L, Elkin B, et al The Arctic as a model for anticipating, preventing, and mitigating climate change impacts on host-parasite interactions. Veterinary Parasitol. 2009;163(3):217–28. 10.1016/j.vetpar.2009.06.008 19560274

[4] Altizer S, Ostfeld RS, Johnson PTJ, Kutz S, Harvell CD. Climate change and infectious diseases: From evidence to a predictive framework. Science. 2013;341(6145):514–9. 10.1126/science.1239401 23908230

[5] Kangbai J, Momoh E. Anthropogenic climatic change risks a global anthrax outbreak: A short communication. J Trop Dis. 2017;5(244):2.

[6] Brearley G, Rhodes J, Bradley A, Baxter G, Seabrook L, Lunney D, et al Wildlife disease prevalence in human-modified landscapes. Biol Rev. 2013;88(2):427–42. 10.1111/brv.12009 23279314

[7] Kutz SJ, Checkley S, Verocai GG, Dumond M, Hoberg EP, Peacock R, et al Invasion, establishment, and range expansion of two parasitic nematodes in the Canadian Arctic. Glob Chang Biol. 2013;19(11):3254–62. 10.1111/gcb.12315 23828740

[8] Atwood TC, Duncan C, Patyk KA, Nol P, Rhyan J, McCollum M, et al Environmental and behavioral changes may influence the exposure of an Arctic apex predator to pathogens and contaminants. Sci Rep. 2017;7(1):13193 10.1038/s41598-017-13496-9 29038498 PMC5643432

[9] Kutz S, Bollinger TK, Branigan M, Checkley S, Davidson R, Dumond M, et al *Erysipelothrix rhusiopathiae* associated with recent widespread muskox mortalities in the Canadian Arcitc. Can Vet J. 2015;6(56):560–3. PMC4431149 26028673

[10] Mascarelli PE, Elmore SA, Jenkins EJ, Alisauskas RT, Walsh M, Breitschwerdt EB, et al Vector-borne pathogens in arctic foxes, Vulpes lagopus, from Canada. Res Vet Sci. 2015;99:58–9. 10.1016/j.rvsc.2014.12.011 25596149

[11] Kock RA, Orynbayev M, Robinson S, Zuther S, Singh NJ, Beauvais W, et al Saigas on the brink: Multidisciplinary analysis of the factors influencing mass mortality events. Sci Adv. 2018;4(1). 10.1126/sciadv.aao2314 PMC5777396 29376120

[12] Jordan PA, Nelson JL, Pastor J. Progress towards the experimental reintroduction of woodland caribou to Minnesota and adjacent Ontario. Rangifer. 1998;18(5):169–81.

[13] Albon SD, Stien A, Irvine RJ, Langvatn R, Ropstad E, Halvorsen O. The role of parasites in the dynamics of a reindeer population. Proc Biol Sci. 2002;269(1500):1625–32. 10.1098/rspb.2002.2064 12184833 PMC1691070

[14] Gulland F. The role of nematode parasites in Soay sheep (*Ovis aries L*.) mortality during a population crash. Parasitology. 1992;105(03):493–503. 1461688 10.1017/s0031182000074679

[15] Pioz M, Loison A, Gauthier D, Gibert P, Jullien J-M, Artois M, et al Diseases and reproductive success in a wild mammal: example in the alpine chamois. Oecologia. 2008;155(4):691–704. 10.1007/s00442-007-0942-5 18189146

[16] Carlsson A, Dobson A, Kutz S. The impact of infectious agents on *Rangifer* populations In: Tryland M, Kutz SJ, editors. Reindeer and Caribou Health and Disease. Boca Raton, FL: CRC Press; 2018 p. 315–52.

[17] Krumm CE, Conner MM, Hobbs NT, Hunter DO, Miller MW. Mountain lions prey selectively on prion-infected mule deer. Biol lett. 2010;6(2):209–11. 10.1098/rsbl.2009.0742 19864271 PMC2865069

[18] Murray DL, Cary JR, Keith LB. Interactive effects of sublethal nematodes and nutritional status on snowshoe hare vulnerability to predation. J Anim Ecol. 1997:250–64.

[19] Stephen C. Toward a modernized definition of wildlife health. J Wildl Dis. 2014;50(3):427–30. 10.7589/2013-11-305 24807179

[20] Ryser-Degiorgis M-P. Wildlife health investigations: needs, challenges and recommendations. BMC Vet Res. 2013;9(1):223. 24188616 10.1186/1746-6148-9-223 PMC4228302

[21] Preece ND, Abell SE, Grogan L, Wayne A, Skerratt LF, Van Oosterzee P, et al A guide for ecologists: Detecting the role of disease in faunal declines and managing population recovery. Biol Conserv. 2017;214:136–46.

[22] Jenkins EJ, Simon A, Bachand N, Stephen C. Wildlife parasites in a One Health world. Trends Parasitol 2015(0). 10.1016/j.pt.2015.01.002 PMC7106350 25662272

[23] Carlsson AM, Veitch A, Popko R, Behrens S, Kutz S. Monitoring wildlife health for conservation and food security in the Canadian Arctic- a case study from the Sahtu settlment area in the Northwest Territories In: Cork SC, Hall DC, Liljebjelke K, editors. One health case studies: addressing complex problems in a changing world Sheffield, UK: 5m Publishing; 2016. p. In review.

[24] Nutall M. Hunting, herding fishing and gathering: Indigenous peoples and renewable resource use in the Arctic. Symon C, Arris L, Grabhorn C, editors. New York: Cambridge University Press; 2005.

[25] Côté SD, Festa-Bianchet M, Dussault C, Tremblay J, Brodeur V, Simard M, et al Caribou herd dynamics: Impacts of climate change on traditional and sport harvesting. Nunavik and Nunatsiavut: From Science to Policy An Integrated Regional Impact Study (IRIS) of Climate Change and Modernization. 2012:249–69.

[26] Vors LS, Boyce MS. Global declines of caribou and reindeer. Glob Chang Biol. 2009;15(11):2626–33.

[27] Festa-Bianchet M, Ray J, Boutin S, Côté S, Gunn A. Conservation of caribou (*Rangifer tarandus*) in Canada: An uncertain future. Can J Zoo. 2011;89(5):419–34.

[28] Mallory CD, Boyce MS. Observed and predicted effects of climate change on Arctic caribou and reindeer. Environ Rev. 2017(999):1–13.

[29] COSEWIC. COSEWIC assessment and status report on the Caribou Rangifer tarandus, Barren-ground population, in Canada. Committee on the Status of Endangered Wildlife in Canada. Ottawa. xiii + 123 pp. (Species at Risk Public Registry website). 2016.

[30] COSEWIC. COSEWIC assessment and status report on the Caribou Rangifer tarandus, Eastern Migratory population and Torngat Mountains population, in Canada. Committee on the Status of Endangered Wildlife in Canada. Ottowa. xvii+ 68pp.; 2017.

[31] COSEWIC. COSEWIC assessment and status report on the Caribou, Dolphin and Union population, Rangifer tarandus, in Canada. Committee on the Status of Endangered Wildlife in Canada. Ottawa. xii + 51 pp; 2017.

[32] Simard A-A, Kutz S, Ducrocq J, Beckmen K, Brodeur V, Campbell M, et al Variation in the intensity and prevalence of macroparasites in migratory caribou: a quasi-circumpolar study. Can J Zoo. 2016;94(9):607–17.

[33] Zarnke RL. Serologic survey for selected microbial pathogens in Alaskan wildlife. J Wildl Dis. 1983;19(4):324–9. 6139490 10.7589/0090-3558-19.4.324

[34] Meyer ME. Identification and virulence studies of Brucella strains isolated from Eskimos and reindeer in Alaska, Canada, and Russia. Am J Vet Res. 1966;27(116):353–8. 4161800

[35] Kutz SJ, Elkin BT, Panay D, Dubey JP. Prevalence of *Toxoplasma gondii* antibodies in barren-ground caribou (*Rangifer tarandus groenlandicus*) from the Canadian Arctic. J Parasitol. 2001;87(2):439–42. 10.1645/0022-3395(2001)087[0439:POTGAI]2.0.CO;2 11318582

[36] Russell DE, Gunn A, White RG. CircumArctic Collaboration to Monitor Caribou and Wild Reindeer. Arctic. 2015;68:6–10.

[37] Kutz S, Ducrocq J, Cuyler C, Elkin B, Gunn A, Kolpashikov L, et al Standardized monitoring of *Rangifer* health during International Polar Year. Rangifer. 2013;33(Sp. Iss. 21):91–114.

[38] Curry PS, Elkin BT, Campbell M, Nielsen K, Hutchins W, Ribble C, et al Filter-paper blood samples for Elisa detection of Brucella antibodies in caribou. J Wildl Dis. 2011;47(1):12–20. 10.7589/0090-3558-47.1.12 21269992

[39] CircumArctic Rangifer Monitoring Assessment Network. Rangifer health and body condition monitoring protocols level 1 and 2: Circumarctic Rangifer Monitoring and Assessment Network; 2008 http://www.caff.is/resources/field-protocols.

[40] Forbes LB. Isolates of Brucella suis biovar 4 from animals and humans in Canada, 1982–1990. Can Vet J. 1991;32(11):686 17423899 PMC1481085

[41] Rhyan JC. Pathogenesis and pathobiology of brucellosis in wildlife. Rev Sci Tech. 2013;32(1):127–36. 23837371 10.20506/rst.32.1.2191

[42] Kutz SJ, Ducrocq J, Verocai GG, Hoar BM, Colwell DD, Beckmen KB, et al Parasites in ungulates of Arctic North America and Greenland: A view of contemporary diversity, ecology, and impact in a world under change. Adv Parasitol. 2012;79:99–252. 10.1016/B978-0-12-398457-9.00002-0 22726643

[43] Bachand N, Ravel A, Leighton P, Stephen C, Ndao M, Avard E, et al Serological and molecular detection of Toxoplasma gondii in terrestrial and marine wildlife harvested for food in Nunavik, Canada. Parasites & vectors. 2019;12(1):155. 30944016 10.1186/s13071-019-3408-9 PMC6448294

[44] Bouchard É, Sharma R, Bachand N, Gajadhar AA, Jenkins EJ. Pathology, clinical signs, and tissue distribution of *Toxoplasma gondii* in experimentally infected reindeer (*Rangifer tarandus*). IJP-PAW. 2017;6(3):234–40. 10.1016/j.ijppaw.2017.08.004 28879089 PMC5573777

[45] Bondo K, Macbeth B, Schwantje H, Orsel K, Culling D, Culling B, et al Health survey of boreal caribou (*Rangifer tarandus caribou*) in northeastern British Columbia, Canada J Wildl Dis.0(0):null. 10.7589/2018-01-018 30605390

[46] Jenkins EJ, Castrodale LJ, de Rosemond SJ, Dixon BR, Elmore SA, Gesy KM, et al Tradition and transition: parasitic zoonoses of people and animals in Alaska, northern Canada, and Greenland. Adv Parasitol. 2013;82:33–204. 10.1016/B978-0-12-407706-5.00002-2 23548085

[47] das Neves CG, Roth S, Rimstad E, Thiry E, Tryland M. Cervid herpesvirus 2 infection in reindeer: A review. Vet Microbiol. 2010;143(1):70–80. 10.1016/j.vetmic.2010.02.015 20207086

[48] Froelich K. Viral diseases of northern ungulates. Rangifer. 2000;20(2–3):83–97.

[49] Tryland M, Neves CGD, Sunde M, Mørk T. Cervid herpesvirus 2, the primary agent in an outbreak of infectious keratoconjunctivitis in semidomesticated reindeer. J Clinic Microbio 2009;47(11):3707–13. 10.1128/JCM.01198-09 PMC2772613 19726598

[50] das Neves CG, Rimstad E, Tryland M. Cervid herpesvirus 2 causes respiratory and fetal infections in semidomesticated Reindeer. J Clinic Microbio. 2009;47(5):1309–13. 10.1128/JCM.02416-08 PMC2681864 19279181

[51] Morton J, Evermann J, Dieterich R. Experimental infection of reindeer with bovine viral diarrhea virus. rangifer. 1990;2(10):75–7.

[52] Lillie LE. The bovine respiratory disease complex. Can Vet J. 1974;15(9):233–42. 4370742 PMC1696627

[53] Palmer MV, Stoffregen WC, Rogers DG, Hamir AN, Richt JA, Pedersen DD, et al West Nile virus infection in reindeer (*Rangifer tarandus*). J Vet Diagn Invest. 2004;16(3):219–22. 10.1177/104063870401600307 15152836

[54] Parkinson AJ, Butler JC. Potential impacts of climate change on infectious diseases in the Arctic. Int J Circumpolar Health. 2005;64(5). 10.3402/ijch.v64i5.18029 16440610

[55] Curry PS. Blood on filter paper for monitoring caribou health: Efficacy, community-based collection and disease ecology in circumpolar herds. Calgary, Alberta: University of Calgary; 2012.

[56] Curry PS, Ribble C, Sears WC, Hutchins W, Orsel K, Godson D, et al Blood collected on filter paper for wildlife serology: Detecting antibodies to *Neospora caninum*, West nile virus, and five bovine viruses in *Rangifer tarandus* subspecies. J Wildl Dis. 2014;50(2):297–307. 10.7589/2012-02-047 24484497

[57] Sergeant, ESG. Epitools epidemiological calculators http://epitools.ausvet.com.au: Ausvet Pty Ltd; 2018 [

[58] Brown LD, Cai TT, DasGupta A. Interval estimation for a binomial proportion. Stat Sci. 2001:101–17.

[59] Heinze G. A comparative investigation of methods for logistic regression with separated or nearly separated data. Stat Med. 2006;25(24):4216–26. 10.1002/sim.2687 16955543

[60] R Core Team. R: A language and environment for statistical computing. R Foundation for Statistical Computing Vienna, Austria: URL http://www.R-project.org/; 2017.

[61] Burnham KP, Anderson DR. Kullback-Leibler information as a basis for strong inference in ecological studies. Wildlife Res. 2001;28(2):111–9.

[62] Arnold TW. Uninformative parameters and model selection using Akaike’s Information Criterion. J Wildl Manag. 2010;74(6):1175–8.

[63] Steele J, Orsel K, Cuyler C, Hoberg EP, Schmidt NM, Kutz SJ. Divergent parasite faunas in adjacent populations of west Greenland caribou: Natural and anthropogenic influences on diversity. IJP-PAW. 2013;2:197–202. 10.1016/j.ijppaw.2013.05.002 24533335 PMC3862502

[64] Hoberg EP, Galbreath KE, Cook JA, Kutz SJ, Polley L. Chapter 1—Northern Host–Parasite Assemblages: History and Biogeography on the Borderlands of Episodic Climate and Environmental Transition In: Rollinson D, Hay SI, editors. Adv Parasitol. 79: Academic Press; 2012 p. 1–97. 10.1016/B978-0-12-398457-9.00001-9 22726642

[65] Yannic G, Pellissier L, Ortego J, Lecomte N, Couturier S, Cuyler C, et al Genetic diversity in caribou linked to past and future climate change. Nat Clim Chang. 2014;4(2):132.

[66] Røed KH. Refugial origin and postglacial colonization of holarctic reindeer and caribou. Rangifer. 2005;25(1):19–30.

[67] Jepsen B, Siegismund HR, Fredholm M. Population genetics of the native caribou (*Rangifer tarandus groenlandicus*) and the semi-domestic reindeer (*Rangifer tarandus tarandus*) in Southwestern Greenland: Evidence of introgression. Conserv Genet. 2002;3(4):401–9.

[68] Stuen S, Krogsrud J, Hyllseth B, Tyler N. Serosurvey of three virus infections in reindeer in northern Norway and Svalbard. Rangifer. 1993;13(4):215–9.

[69] Gilbert AT, Fooks AR, Hayman DT, Horton DL, Müller T, Plowright R, et al Deciphering serology to understand the ecology of infectious diseases in wildlife. EcoHealth. 2013;10(3):298–313. 10.1007/s10393-013-0856-0 23918033

[70] Tomaselli M, Kutz S, Gerlach C, Checkley S. Local knowledge to enhance wildlife population health surveillance: Conserving muskoxen and caribou in the Canadian Arctic. Biol Conserv. 2018;217:337–48.

[71] Tryland M, Kutz SJ. Reindeer and Caribou, Health and Disease. Boca Raton, FL: CRC Press; 2018.

[72] Campbell M. Research update to the Department of Environment: population estimate of a declining population of island bound barren-ground caribou (*Rangifer tarandus groenlandicus*), Southhampton Island, NU Department of Environment, Kivalliq Region; 2013.

[73] Ferguson MA. Rangiferine brucellosis on Baffin Island. J Wildl Dis. 1997;33(3):536–43. 10.7589/0090-3558-33.3.536 9249700

[74] Gunn A, Leighton T, Wobeser G. Wildlife diseases and parasites in the Kitkmeot region, 1984–90. Department of Renewable Resources, Government of the Northwest Territories; 1991.

[75] Zarnke R. Serologic survey of alaska wildlife for microbial pathogens. In: Game ADoFa, editor. Juneau, AK 1999 p. 18pp.

[76] Kutz SJ, Ducrocq J, Verocai GG, Hoar BM, Colwell DD, Beckmen KB, et al Parasites in ungulates of Arctic North America and Greenland: A view of contemporary diversity, ecology, and impact in a world under change. Advances in Parasitology. 2012;79:99–252. 10.1016/B978-0-12-398457-9.00002-0 22726643

[77] Stieve E, Beckmen K, Kania SA, Widner A, Patton S. *Neospora caninum* and *Toxoplasma gondii* antibody prevalence in Alaska wildlife. J Wildl Dis. 2010;46(2):348–55. 10.7589/0090-3558-46.2.348 20688628

[78] Zarnke RL, Dubey J, Ver Hoef J, McNay M, Kwok O. Serologic survey for *Toxoplasma gondii* in lynx from interior Alaska. J Wildl Dis. 2001;37(1):36–8. 10.7589/0090-3558-37.1.36 11272502

[79] Duong MC, Alenius S, Huong LTT, Björkman C. Prevalence of *Neospora caninum* and bovine viral diarrhoea virus in dairy cows in Southern Vietnam. Vet J. 2008;175(3):390–4. 10.1016/j.tvjl.2006.01.016 17349807

[80] Björkman C, Alenius S, Manuelsson U, Uggla A. *Neospora caninum* and Bovine Virus Diarrhoea Virus Infections in Swedish Dairy Cows in Relation to Abortion. Vet J. 2000;159(2):201–6. 10.1053/tvjl.1999.0446 10712809

[81] Bartels C, Wouda W, Schukken Y. Risk factors for *Neospora caninum*-associated abortion storms in dairy herds in The Netherlands (1995 to 1997). Theriogenology. 1999;52(2):247–57. 10.1016/S0093-691X(99)00126-0 10734392

[82] Ståhl K, Björkman C, Emanuelson U, Rivera H, Zelada A, Moreno-López J. A prospective study of the effect of *Neospora caninum* and BVDV infections on bovine abortions in a dairy herd in Arequipa, Peru. Prev Vet Med. 2006;75(3):177–88. 16597470 10.1016/j.prevetmed.2006.02.006

[83] Quinn HE. An outbreak of abortion in a dairy herd associated with *Neospora caninum* and bovine pestivirus infection. Aus Vet J. 2004;82(1‐2):99–101. 10.1111/j.1751-0813.2004.tb14656.x 15088970

[84] Elazhary MASY, Frechette JL, Silim A, Roy RS. Serological evidence of some bovine viruses in the caribou (*Rangifer tarandus caribou*) in Quebec. J Wildl Dis. 1981;17(4):609–12. 6121921 10.7589/0090-3558-17.4.609

[85] Evans AL, das Neves CG, Finstad GF, Beckmen KB, Skjerve E, Nymo IH, et al Evidence of alphaherpesvirus infections in Alaskan caribou and reindeer. BMC Vet Res. 2012;8. 10.1186/1746-6148-8-5 PMC3274481 22243919

[86] Johnson D, Harms NJ, Larter NC, Elkin BT, Tabel H, Wei G. Serum biochemistry, serology, and parasitology of boreal caribou (*Rangifer tarandus caribou*) in the Northwest Territories, Canada. J Wildl Dis. 2010;46(4):1096–107. 10.7589/0090-3558-46.4.1096 20966261

[87] Kautto AH, Alenius S, Mossing T, Becher P, Belák S, Larska M. Pestivirus and alphaherpesvirus infections in Swedish reindeer (*Rangifer tarandus tarandus L*.). Vet Microbiol. 2012;156(1):64–71. 22078277 10.1016/j.vetmic.2011.10.018

[88] Lillehaug A, Vikøren T, Larsen I-L, Åkerstedt J, Tharaldsen J, Handeland K. Antibodies to ruminant alpha-herpesviruses and pestiviruses in norwegian cervids. J Wildl Dis. 2003;39(4):779–86. 10.7589/0090-3558-39.4.779 14733272

[89] Rehbinder C, Belák S, Nordkvist M. A serological, retrospective study in reindeer on five different viruses. Rangifer. 1992;12(3):191–5.

[90] Jordan LT, Rettie WJ, Tessaro SV. Evidence of herpesvirus infection in woodland caribou in Saskatchewan. J Wildl Dis. 2003;39(1):216–20. 10.7589/0090-3558-39.1.216 12685086

[91] das Neves CG, Roger M, Yoccoz NG, Rimstad E, Tryland M. Evaluation of three commercial bovine ELISA kits for detection of antibodies against Alphaherpesviruses in reindeer (Rangifer tarandus tarandus). Acta Vet Scand. 2009;51. 19272136 10.1186/1751-0147-51-9 PMC2663558

[92] Avalos-Ramirez R, Orlich M, Thiel H-J, Becher P. Evidence for the presence of two novel pestivirus species. Virology. 2001;286(2):456–65. 10.1006/viro.2001.1001 11485413

[93] Larska M. Pestivirus infection in reindeer (*Rangifer tarandus*). Front MicroBio. 2015;6. 10.3389/fmicb.2015.01187 PMC4620691 26579094

[94] Ek-Kommonen C. Isolation of a herpesvirus serologically related to bovine herpesvirus 1 from a reindeer (Rangifer tarandus). Acta Veterinaria Scandinavica. 1986;27(2):299–301. 3026156 10.1186/BF03548174 PMC8189397

[95] Rockborn G, Rehbinder C, Klingeborn B, Leffler M, Klintevall K, Nikkilä T, et al The demonstration of a herpesvirus, related to bovine herpesvirus 1, in reindeer with ulcerative and necrotizing lesions of the upper alimentary tract and nose. Rangifer. 1990;10(3):373–84.

[96] das Neves CG, Mørk T, Thiry J, Godfroid J, Rimstad E, Thiry E, et al Cervid herpesvirus 2 experimentally reactivated in reindeer can produce generalized viremia and abortion. Virus Res. 2009;145(2):321–8. 10.1016/j.virusres.2009.08.002 19699769

[97] das Neves CG, Thiry J, Skjerve E, Yoccoz NG, Rimstad E, Thiry E, et al Alphaherpesvirus infections in semidomesticated reindeer: a cross-sectional serological study. Vet Microbiol. 2009;139(3):262–9. 19604658 10.1016/j.vetmic.2009.06.013

[98] Lindberg ALE. Bovine viral diarrhoea virus infections and its control—A review. Vet Q. 2003;25(1):1–16. 10.1080/01652176.2003.9695140 12670010

[99] Nettleton P, Entrican G. Ruminant pestiviruses. Br Vet J. 1995;151(6):615–42. 8605577 10.1016/S0007-1935(95)80145-6 PMC7130397

[100] Tryland M, Mørk T, Ryeng KA, Sørensen KK. Evidence of parapox-, alphaherpes-and pestivirus infections in carcasses of semi-domesticated reindeer (*Rangifer tarandus tarandus*) from Finnmark, Norway. Rangifer. 2005;25(2):75–83.

[101] Brackenbury L, Carr B, Charleston B. Aspects of the innate and adaptive immune responses to acute infections with BVDV. Vet Microbiol. 2003;96(4):337–44. 14599781 10.1016/j.vetmic.2003.09.004

[102] Biswas S, Bandyopadhyay S, Dimri U, Patra PH. Bovine herpesvirus-1 (BHV-1)—a re-emerging concern in livestock: a revisit to its biology, epidemiology, diagnosis, and prophylaxis. Vet Q. 2013;33(2):68–81. 10.1080/01652176.2013.799301 23802762

[103] Ridpath J. The Contribution of Infections with Bovine Viral Diarrhea Viruses to Bovine Respiratory Disease. Vet Clin North Am Food Anim Pract. 2010;26(2):335–48. 10.1016/j.cvfa.2010.04.003 20619188

[104] Molina V, Risalde MA, Sánchez-Cordón PJ, Pedrera M, Romero-Palomo F, Luzzago C, et al Effect of infection with BHV-1 on peripheral blood leukocytes and lymphocyte subpopulations in calves with subclinical BVD. Res Vet Sci. 2013;95(1):115–22. 10.1016/j.rvsc.2013.02.018 23541923

[105] Edwards S, Wood L, Hewitt-Taylor C, Drew TW. Evidence for an immunocompromising effect of bovine pestivirus on bovid herpesvirus 1 vaccination. Vet Res Commun. 1986;10(1):297–302. 3016976 10.1007/BF02213992

[106] Risalde MA, Molina V, Sonchez-Cordon PJ, Pedrera M, Romero-Palomo F, Bautista MJ, et al Comparison of pathological changes and viral antigen distribution in tissues of calves with and without preexisting bovine viral diarrhea virus infection following challenge with bovine herpesvirus-1. Am J Vet Res. 2013;74(4):598–610. 10.2460/ajvr.74.4.598 23531068

[107] Hellard E, Fouchet D, Vavre F, Pontier D. Parasite-parasite interactions in the wild: How to detect them? Trends Parasitol. 2015;31(12):640–52. 10.1016/j.pt.2015.07.005 26440785

[108] Rigaud T, Perrot-Minnot M-J, Brown MJ. Parasite and host assemblages: embracing the reality will improve our knowledge of parasite transmission and virulence. Proc R Soc London B Bio. 2010;277(1701):3693–702. 10.1098/rspb.2010.1163 PMC2992712 20667874

[109] Telfer S, Lambin X, Birtles R, Beldomenico P, Burthe S, Paterson S, et al Species interactions in a parasite community drive infection risk in a wildlife population. Science. 2010;330(6001):243–6. 10.1126/science.1190333 20929776 PMC3033556

[110] Alizon S, de Roode JC, Michalakis Y. Multiple infections and the evolution of virulence. Ecol Lett. 2013;16(4):556–67. 10.1111/ele.12076 23347009

[111] Campbell JR. Effect of bovine viral diarrhea virus in the feedlot. Vet Clin North Am Food Anim Pract. 2004;20(1):39–50. 10.1016/j.cvfa.2003.11.003 15062473 PMC7119114

[112] Pedersen AB, Fenton A. The role of antiparasite treatment experiments in assessing the impact of parasites on wildlife. Trends Parasitol. 2015;31(5):200–11. 10.1016/j.pt.2015.02.004 25778845

[113] Van der Poel WHM, Langedijk JPM, Kramps JA, Middel WGJ, Brand A, Van Oirschot JT. Bovine respiratory syncytial virus antibodies in non-bovine species. Arch Virol. 1995;140(9):1549–55. 7487487 10.1007/BF01322529

[114] Blanchong JA, Robinson SJ, Samuel MD, Foster JT. Application of genetics and genomics to wildlife epidemiology. J Wildl Manag. 2016;80(4):593–608.

[115] Lanyon SR, Hill FI, Reichel MP, Brownlie J. Bovine viral diarrhoea: pathogenesis and diagnosis. Vet J. 2014;199(2):201–9. 10.1016/j.tvjl.2013.07.024 24053990

[116] Tryland M, Romano JS, Marcin N, Nymo IH, Josefsen TD, Sørensen KK, et al Cervid herpesvirus 2 and not Moraxella bovoculi caused keratoconjunctivitis in experimentally inoculated semi-domesticated Eurasian tundra reindeer. Acta Vet Scand. 2017;59(1):23 10.1186/s13028-017-0291-2 28438213 PMC5404682

[117] Grissett G, White B, Larson R. Structured literature review of responses of cattle to viral and bacterial pathogens causing bovine respiratory disease complex. J Vet Intern Med. 2015;29(3):770–80. 10.1111/jvim.12597 25929158 PMC4895424

[118] Dubey JP, Jenkins MC, Rajendran C, Miska K, Ferreira LR, Martins J, et al Gray wolf (*Canis lupus*) is a natural definitive host for *Neospora caninum*. Vet Parasitol. 2011;181(2–4):382–7. 10.1016/j.vetpar.2011.05.018 21640485

[119] Dubey J, Lewis B, Beam K, Abbitt B. Transplacental toxoplasmosis in a reindeer (Rangifer tarandus) fetus. Vet Parasitol. 2002;110(1–2):131–5. 12446098 10.1016/s0304-4017(02)00320-5

[120] Oksanen A, Gustafsson K, Lundén A, Dubey J, Thulliez P, Uggla A. Experimental Toxoplasma gondii infection leading to fatal enteritis in reindeer (*Rangifer tarandus*). J Parasitol. 1996:843–5. 8885901

[121] Davis LE, DeBiasi R, Goade DE, Haaland KY, Harrington JA, Harnar JB, et al West Nile virus neuroinvasive disease. Annal Neurol. 2006;60(3):286–300. 10.1002/ana.20959 16983682

[122] Durham PJ, Hassard LE. Prevalence of antibodies to infectious bovine rhinotracheitis, parainfluenza 3, bovine respiratory syncytial, and bovine viral diarrhea viruses in cattle in Saskatchewan and Alberta. Can Vet J. 1990;31(12):815 17423704 PMC1480901

[123] Gall D, Nielsen K. Serological diagnosis of bovine brucellosis: a review of test performance and cost comparison. Rev Sci Tech. 2004;23(3):989–1002. 15861895

[124] Nymo IH, Godfroid J, Asbakk K, Larsen AK, das Neves CG, Rodven R, et al A protein A/G indirect enzyme-linked immunosorbent assay for the detection of anti-Brucella antibodies in Arctic wildlife. J Vet Diagn Invest. 2013;25(3):369–75. 10.1177/1040638713485073 23572454

[125] Dubey J, Desmonts G. Serological responses of equids fed Toxoplasma gondii oocysts. Equine Vet J. 1987;19(4):337–9. 3622463 10.1111/j.2042-3306.1987.tb01426.x

[126] Blitvich BJ, Bowen RA, Marlenee NL, Hall RA, Bunning ML, Beaty BJ. Epitope-blocking enzyme-linked immunosorbent assays for detection of West Nile virus antibodies in domestic mammals. J Clin Microbiol. 2003;41(6):2676–9. 10.1128/JCM.41.6.2676-2679.2003 12791902 PMC156482

