# Supplementary material for: Multi-pathogen serological survey of migratory caribou herds: A snapshot in time
Source: PLoS One. 2019 Jul 31;14(7):e0219838. doi: 10.1371/journal.pone.0219838 (PMC6668789; doi:10.1371/journal.pone.0219838)
Supplement: S1 Table — Summary of seasons and years sample collection occurred for each herd, including the type of collection. CARMA IPY refers to scientific collections conducted during the International Polar Years by the CircumArctic Rangifer Monitoring Assessment Network. (PDF) [file pone.0219838.s001.pdf]

Herd	Collection Type	Season/Years
Porcupine	CARMA IPY	Fall 08, 09<br>Summer, 09
Bluenose West	CARMA IPY<br>Subsistence hunters<br>Community hunts	Fall 07, 08, 13<br>Spring 05, 06, 10, 14<br>Winter 05, 06, 10, 14
Bluenose East	Community based monitoring program<br>Community hunts	Spring 04-07, 09, 13, 14<br>Summer 08<br>Winter 05, 08, 13, 14
Dolphin and Union	Collaring events<br>Scientific collections	Spring 15, 16
Bathurst	CARMA IPY	Fall 07, 08<br>Spring 08, 09
Beverly and Ahiak	Collaring events<br>Community based sampling	Spring 00, 06, 07, 08, 12, 14<br>Summer 07<br>Winter 09
Quaminuriaq	Scientific collections	Fall 08-10<br>Spring 08-10<br>Summer 08-10<br>Winter 09,10
Rivière-aux-Feuilles	Caribou Ungava<br>CARMA IPY	Fall 07, 08, 09<br>Summer 07, 08, 09
Rivière-George	Caribou Ungava<br>CARMA IPY	Fall 07, 08, 09<br>Summer 07, 08, 09
Akia-Maniitsoq	CARMA IPY	Spring 08
Kangerlussuaq-Sisimiut	CARMA IPY	Spring 09
