# Supplementary material for: Multi-pathogen serological survey of migratory caribou herds: A snapshot in time
Source: PLoS One. 2019 Jul 31;14(7):e0219838. doi: 10.1371/journal.pone.0219838 (PMC6668789; doi:10.1371/journal.pone.0219838)
Supplement: S3 Table — Observed sample seroprevalence of screened pathogens for calf female (F) and male (M) caribou, and overall (O) by herd. Pathogen abbreviations: Alphaherpesvirus (Herp), Pestivirus (Pesti), Parainfluenzavirus type 3 (PI3), Neospora caninum (Neo), Brucella suis biovar 4 (Bru), Toxoplama gondii (Toxo), West Nile Virus (WNV), Bovine respiratory syntical virus (BRSV). Caribou herd abbreviations: Porcupine (PCH), Bluenose West (BNW), Bluenose East (BNE), Dolphin and Union (DU), Bathurst (BA), Beverly and Ahiak (BEAH), Quaminuriaq (QAM), Rivière-aux-Feuilles (R-F), Rivière-George (R-G), Akia-Maniitsoq (AK) and Kangerlussuaq-Sisimiut (KA). Herds are listed west to east geographically left to right. Sample seroprevalence (%), number of positive samples (p), sample size (n), 95% Clopper-Pearson Exact confidence intervals (CI) and number of doubtful samples (D) are presented. (PDF) [file pone.0219838.s003.pdf]

S4		BNW	BNE	BA			QAM			G-F			G-R			AK	KA			ALL HERDS		
		F	F	F	M	O	F	M	O	F	M	O	F	M	O	F	F	M	O	F	M	O
**Herp**	%	0	0	11	0	12	33	0	25	0	0	0	0	0	0	0	0	0	0	4	0	3
	CI	0-98	0-84	3-48	0-60	8-19	1-91	0-98	1-81	0-12	0-20	0-8	0-16	0-22	0-10	0-46	0-46	0-60	0-31	0-9	0-9	0-6
	p	0	0	1	0	1	1	0	1	0	0	0	0	0	0	0	0	0	0	2	0	2
	n	1	2	9	4	13	3	1	4	28	17	45	21	15	36	6	6	4	10	76	41	117
	D	0	0	0	0	0	0	0	0	0	0	0	0	0	0	0	0	0	0	0	0	0
**Pesti**	%	0	100	29	33	30	33	0	25	33	25	30	75	50	67	0	0	25	10	35	32	34
	CI	0-98	3-100	4-71	1-91	7-65	1-91	0-98	1-81	10-65	3-65	12-54	43-95	12-88	41-87	0-46	0-46	1-81	0-45	22-51	14-55	23-47
	p	0	1	2	1	3	1	0	1	4	2	6	9	3	12	0	0	1	1	17	7	24
	n	1	1	7	3	10	3	1	4	12	8	20	12	6	18	6	6	4	10	48	22	70
	D	0	1	2	1	3	0	0	0	16	9	25	11	12	23	0	0	0	0	30	22	52
**PI3**	%	0	0	0	0	0	0	0	0	0	0	0	0	0	0	0	0	0	0	0	0	0
	CI	0-98	0-84	0-34	0-60	0-25	0-71	0-98	0-60	0-12	0-20	0-8	0-16	0-22	0-10	0-46	0-46	0-60	0-31	0-5	0-9	0-3
	p	0	0	0	0	0	0	0	0	0	0	0	0	0	0	0	0	0	0	0	0	0
	n	1	2	9	4	13	3	1	4	28	17	45	21	15	36	6	6	4	10	76	41	117
**Neo**	%	-	-	0	0	0	100	100	100	0	0	0	0	0	0	0	0	0	0	8	5	0
	CI	-	-	0-34	0-71	0-27	29-100	3-100	40-100	0-41	0-41	0-23	0-41	0-37	0-22	0-46	0-46	0-71	0-34	2-20	0-23	2-16
	p	-	-	0	0	0	3	1	4	0	0	0	0	0	0	0	0	0	0	3	1	4
	n	-	2	9	3	12	3	1	4	7	7	14	7	8	15	6	6	3	9	40	22	62
**Bru**	%	0	0	0	0	0	0	0	0	0	0	0	0	0	0	0	0	0	0	0	0	0
	CI	0-98	0-84	0-34	0-60	0-25	0-71	0-98	0-60	0-12	0-20	0-8	0-15	0-19	0-9	0-46	0-46	0-60	0-31	0-5	0-8	0-3
	p	0	0	0	0	0	0	0	0	0	0	0	0	0	0	0	0	0	0	0	0	0
	n	1	2	9	4	13	3	1	4	28	17	45	23	18	41	6	6	4	10	78	44	122
**Toxo**	%	0	0	0	0	0	0	-	-	0	0	0	4	0	3	6	0	0	0	1	0	1
	CI	0-98	0-98	0-34	0-60	0-25	0-98	-	-	0-12	0-20	0-8	0-22	0-19	0-13	0-46	0-46	0-60	0-31	0-7	0-8	0-5
	p	0	0	0	0	0	0	-	-	0	0	0	1	0	1	0	0	0	0	1	0	1
	n	1	2	9	4	13	1	-	-	29	17	46	23	18	41	0	6	4	10	77	43	120
	D	0	0	0	0	0	0	-		0	0	0	0	0	0	0	0	0	0	0	0	0
**WNV**	%	-	-	0	0	0	-	-	-	0	0	0	0	0	0	0	0	0	0	0	0	0
	CI	-	-	0-34	0-60	0-25	-	-	-	0-12	0-20	0-8	0-15	0-19	0-9	0-46	0-46	0-60	0-31	0-5	0-8	0-3
	p	-	-	0	0	0	-	-	-	0	0	0	0	0	0	0	0	0	0	0	0	0
	n	-	-	9	4	13	-	-	-	28	17	45	23	18	41	6	6	4	10	72	43	115
