# Supplementary material for: Multi-pathogen serological survey of migratory caribou herds: A snapshot in time
Source: PLoS One. 2019 Jul 31;14(7):e0219838. doi: 10.1371/journal.pone.0219838 (PMC6668789; doi:10.1371/journal.pone.0219838)
Supplement: S4 Table — Summary of top 10 models, based on ΔAICc, predicting kidney fat index and backfat in caribou, where K is the number of parameters, wi is the model Akaike weight and LL is the log-likelihood. The model used for inference is highlighted in grey. Explanatory variables included were: Herd, Season (Summer, Fall), Tage (age as determined by cementum tooth age analysis), Sex, Co-exposure (alphaherpesvirus (Herp) and/or, esPestivirus serostatus) (positive, negative)) and sampling year. (PDF) [file pone.0219838.s004.pdf]

**Body Condition**	**Models**	**K**	**AICc**	**ΔAICc**	***wᵢ***	**LL**
Kidney Fat Index	1k. Herd+Seas+Tage+Tage^2	6	693.77	0.00	0.25	-340.50
	2k. Herd+Seas+Herp+Tage+Tage^2	7	694.45	0.68	0.18	-339.71
	3k. Herd+Seas+Tage	5	695.51	1.74	0.11	-342.48
	4k. Herd+Seas+Pesti+Tage+Tage^2	7	695.97	2.20	0.08	-340.47
	5k. Herd+Seas+Pesti+Herp+Tage+Tage^2	8	696.43	2.66	0.07	-339.54
	6k. Herd+Seas+Year+Tage+Tage^2	8	696.86	3.09	0.05	-339.76
	7k. Herd+Seas+Herp+Tage	6	697.13	3.36	0.05	-342.18
	8k. Herd+Seas+Pesti+Tage	6	697.63	3.86	0.04	-342.43
	9k. Herd+Seas	4	697.83	4.06	0.03	-344.73
	10k. Herd+Seas+Year+Tage	7	698.66	4.89	0.02	-341.81
Back Fat	1b. Herd	3	361.22	0	0.29	-177.39
	2b. Herd+Herpes	4	363.16	1.94	0.11	-177.20
	3b. Herd+YearF	5	363.34	2.12	0.1	-176.09
	4b. BF+Tage	4	363.45	2.24	0.09	-177.35
	5b. Herd+Pesti	4	363.47	2.25	0.09	-177.36
	6b. Herd+Herpes+Year	6	365.22	4	0.04	-175.79
	7b. Herd+Herpes+Tage	5	365.36	4.14	0.04	-177.10
	8b. Herd+Pesti+Herpes	5	365.44	4.22	0.04	-177.14
	9b. Herd+Pesti+Year	6	365.76	4.54	0.03	-176.06
	10b. Herd+Pesti+Tage	5	365.81	4.59	0.03	-177.33
