# Supplementary material for: Multi-pathogen serological survey of migratory caribou herds: A snapshot in time
Source: PLoS One. 2019 Jul 31;14(7):e0219838. doi: 10.1371/journal.pone.0219838 (PMC6668789; doi:10.1371/journal.pone.0219838)
Supplement: S2 Table — Observed sample seroprevalence of screened pathogens for yearling female (F) and male (M) caribou, and overall (O) by herd. Pathogen abbreviations: Alphaherpesvirus (Herp), Pestivirus (Pesti), Parainfluenzavirus type 3 (PI3), Neospora caninum (Neo), Brucella suis biovar 4 (Bru), Toxoplama gondii (Toxo), West Nile Virus (WNV), Bovine respiratory syntical virus (BRSV). Caribou herd abbreviations: Porcupine (PCH), Bluenose West (BNW), Bluenose East (BNE), Dolphin and Union (DU), Bathurst (BA), Beverly and Ahiak (BEAH), Quaminuriaq (QAM), Rivière-aux-Feuilles (R-F), Rivière-George (R-G), Akia-Maniitsoq (AK) and Kangerlussuaq-Sisimiut (KA). Herds are listed west to east geographically left to right. Sample seroprevalence (%), number of positive samples (p), sample size (n), 95% Clopper-Pearson Exact confidence intervals (CI) and number of doubtful samples (D) are presented. (PDF) [file pone.0219838.s002.pdf]

S3		BNW			BNE	BA			BEAH			QAM			LEA	ALL HERDS		
		F	M	O	M	F	M	O	F	M	O	F	M	O	F	F	M	O
**Herp**	%	0	33	25	33	25	0	0	80	100	83	14	14	14	0	32	24	28
	CI	0-84	4-78	3-65	1-91	1-81	0-60	0-53	28-99	3-100	36-100	0-58	0-58	2-43	0-98	13-57	8-47	15-44
	p	0	2	2	1	1	0	1	4	1	5	1	1	2	0	6	5	11
	n	2	6	8	3	4	4	8	5	1	6	7	7	14	1	19	21	40
	D	0	0	0	0	0	0	0	0	0	0	0	0	0	0	0	0	0
**Pesti**	%	0	17	13	-	33	50	40	0	0	0	0	0	0	0	6	14	9
	CI	0-84	0-64	0-53	-	1-91	1-99	5-85	0-52	0-98	0-46	0-41	0-41	0-23	0-98	0-27	2-43	2-25
	p	0	1	1	-	1	1	2	0	0	0	0	0	0	0	1	2	3
	n	2	4	6	-	3	2	5	5	1	6	7	7	14	1	18	14	32
	D	0	2	2	3	1	2	3	0	0	0	0	0	0	0	1	7	8
**PI3**	%	0	33	25	67	0	0	0	0	0	0	0	0	0	0	0	19	10
	CI	0-84	4-78	3-65	9-99	0-60	0-60	0-37	0-52	0-98	0-46	0-41	0-41	0-23	0-98	0-18	5-42	3-24
	p	0	2	2	2	0	0	0	0	0	0	0	0	0	0	0	4	4
	n	2	6	8	3	4	4	8	5	1	6	7	7	14	1	19	21	40
**Neo**	%	0	-	0	-	0	0	0	20	100	33	100	100	100	-	44	73	56
	CI	0-98	-	0-98	-	0-60	0-71	0-41	1-72	3-100	4-78	54-100	59-100	75-100	-	20-70	39-94	35-75
	p	0	-	0	-	0	0	0	1	1	2	6	7	13	-	7	8	15
	n	1	-	1	-	4	3	7	5	1	6	6	7	13	-	16	11	27
**Bru**	%	0	0	0	0	0	0	0	0	0	0	0	0	0	0	0	0	0
	CI	0-71	0-41	0-31	0-71	0-52	0-60	0-34	0-52	0-98	0-46	0-41	0-41	0-23	0-98	0-16	0-15	0-8
	p	0	0	0	0	0	0	0	0	0	0	0	0	0	0	0	0	0
	n	3	7	10	3	5	4	9	5	1	6	7	7	14	1	21	22	43
**Toxo**	%	0	0	0	0	25	0	12.5	0	0	0	0	0	0	0	7	0	3
	CI	0-71	0-41	0-31	0-71	1-81	0-60	0-53	0-52	0-98	0-46	0-98	0-84	0-71	0-98	0-34	0-19	0-17
	p	0	0	0	0	1	0	1	0	0	0	0	0	0	0	1	0	1
	n	3	7	10	3	4	4	8	5	1	6	1	2	3	1	14	17	31
	D	0	0	0	0	0	0	0	0	0	0	0	0	0	0	0	0	0
**WNV**	%	0	0	0	-	0	0	0	-	-	-	-	-	-	0	0	0	0
	CI	0-98	0-98	0-84	-	0-60	0-60	0-40	-	-	-	-	-	-	0-90	0-46	0-52	0-29
	p	0	0	0	-	0	0	0	-	-	-	-	-	-	0	0	0	0
	n	1	1	2	-	4	4	8	-	-	-	-	-	-	1	6	5	11
**BRSV**	%	0	0	0	-	0	0	0	-	-	-	-	-	-	0	0	0	0
	CI	0-98	0-98	0-84	-	0-60	0-60	0-40	-	-	-	-	-	-	0-90	0-46	0-52	0-29
	p	0	0	0	-	0	0	0	-	-	-	-	-	-	0	0	0	0
	n	1	1	2	-	4	4	8	-	-	-	-	-	-	1	6	5	11
